# Biological characteristics and genetics differentiation of smut fungi in *Coix* L.

**DOI:** 10.3389/fmicb.2026.1746178

**Published:** 2026-04-17

**Authors:** Chuanqi Zheng, Fuhai Peng, Meilin Linghu, Xiangdong Li, Dali Sun, Panpan Yang

**Affiliations:** 1 Qianxinan Research Institute of Agricultural and Forestry Sciences, Xingyi, Guizhou, China; 2 Institute of Resources and Environment, Guizhou University, Guiyang, Guizhou, China; 3 School of Public Health, Guizhou Medical University, Guiyang, China; 4 State Key Laboratory of Vegetable Biobreeding, Institute of Vegetables and Flowers, Chinese Academy of Agricultural Sciences, Beijing, China

**Keywords:** adlay smut, biological characteristics, genome sequencing, morphology, nutritional value, teliospores development

## Abstract

Adlay is one of the most important crops of subtropical regions, and it can be affected by several fungal pathogens, with adlay smut being one of the most devastating diseases. In this paper, two smut fungi strains, ZC202104 and ZC202205, were isolated from infected plants and identified as *Ustilago coicis* based on morphology and multi-gene phylogenetic analysis (ITS, LSU, ATP6). The two strains showed distinct genetic differentiation in morphology characteristics. ZC202205 exhibited a unique off-white colony with central fluff and lacked yeast-like folds, a feature not previously reported. Moreover, the two strains also differed in cultural features and mycelial nutrient composition. Genome sequencing revealed similar genome sizes (~22 Mb) and GC content (~53.8%) for both strains, with thousands of interspersed and tandem repeats. Cluster analysis of homologous gene families encompassed 10 species of smut fungi strains, with encoding gene counts ranging from 5,756 to 6,783. The phylogenetic clustering annalysis revealed that the *Ustilago* genus diverged into two clades approximately 109.2 million years ago (MYA) and *Sporisorium* genus were further diverged from *Ustilago* in about 103 million years ago. *Ustilago coicis* clustered with *Ustilago trichophora*, indicating a closer phylogenetic relationship. Gene family dynamics analysis revealed a significant trend of gene expansion in *Ustilago coicis*, with few genes contracted. This study integrates the biological characteristics and genomic data of *Ustilago coicis* to enhance the understanding of its evolutionary status, providing a foundation for elucidating the infection mechanisms of *Ustilago coicis* as well as its development and utilization.

## 1 Introduction

Adlay (*Coix lacryma-jobi* L.), also known as Job’s tears, is a grass crop belonging to the gramineae family that has been traditionally cultivated for its medicinal and dietary purposes. It is renowned as “the king of Gramineae in the world” (Yang, 1993; Chang et al., 2003). Originating from subtropical countries, adlay are extensively grown in Asian nations such as China, Japan, Thailand, and South Korea (Wu et al., 2024). Guizhou, Guangxi, Yunnan, and other areas in southwestern and southern China are primary centers for the origin, evolution, and migration of adlay. Adlay is one of the main cash crops in Guizhou Province, China. It covers a national planting area of about 80,000 hectares, with 32,000 hectares planted in Guizhou. In Xingren City, Guizhou, the planting area of adlay is 16,700 hectares, with a comprehensive annual output value of approximately 2.6 billion yuan across the primary, secondary, and tertiary industries (Diao, 2017; Zhang, 2024). The dried and mature seeds known as coicis semen are considered as the homology of medicine and food due to their nutritional value and high pharmacological activity. Coix seeds contain various chemical constituents including fatty acids, esters, sterols, polysaccharides, flavonoids and lignans (Wu et al., 2023), and has historically been used to relieve edema, dysuria, and diarrhea (Chinese Pharmacopoeia Commission, 2020). In modern pharmacological research, diverse medicinal properties of coicis semen had been demonstrated including natural anti-tumor effects, treatment of hypoglycemia, regulation of intestinal microbiota, improvement of liver function, and anti-inflammatory effects (Weng et al., 2022). Moreover, it can be consumed as food and utilized in soup, pills, powders, wine and congee preparations (Wang, 2020). Therefore, adlay had immense potential for development and utilization.

Adlay smut is one of the most common and important diseases that greatly affects the quality and yield of adlay. The smut genera *Ustilago*, *Sporisorium*, and *Macalpinomyces* (*Basidiomycota*: *Ustilaginales*: *Ustilaginaceae*) are systemic diseases on grasses plant (Gramineae). These fungi are typically dimorphic, producing haploid spores for asexual reproduction and dikaryotic hyphae for sexual reproduction (Bakkeren et al., 2008; Begerow et al., 2006). They exhibit host specificity toward parasitic crops, for instance, *Sporisorium scitamineum* specifically parasitizes sugarcane, while *Sporisorium reilianum* targets sorghum (Tanaka et al., 2019). The causal agent of adlay smut was named *Ustilago coicis* Bref., as identified by Small. This pathogen can infect adlay, resulting in damage to its stems and leaves, and ultimately leading to the formation of tumors on the aboveground parts of the plant (Small, 1927). The infection is particularly evident in continuous cropping, resulting in a 30 to 80% reduction in adlay yield, which significantly impacts the sustainable development of adlay (Luo et al., 2019). Currently, reports on adlay smut mainly focused on field observations and incidence control (Zhou et al., 2014; Liu and Shen, 2016). Few studies had explored the genetic differentiation, biological characteristics of mycelium as well as the growth, development, and genetic basis of *Ustilago coicis*.

With the advancement of molecular biology, microbial genome sequencing has also developed rapidly, particularly in the field of plant pathogenic fungi. The completion of pathogenic fungal genome sequencing has promoted the study of pathogenic fungal population genomics, which is of great significance for studying population structure, evolutionary mechanisms, and the discovery of key genes. Utilizing genomic methods not only allows for in-depth analysis of pathogenic fungal population structure and genetic diversity, providing a theoretical basis for the prevention of plant disease epidemics, but also lays the foundation for the study of genetic diversity related to the biosynthesis of secondary metabolites (Zhen, 2024). In previous studies, *Ustilago* and *Sporisorium* fungal taxa were detected in the sori of adlay smut using ITS metagenomic sequencing (Li et al., 2021). Subsequently, a purified strain was successfully isolated from the sori of adlay smut, and its genome was assembled (Li et al., 2022). Based on the research above, it is speculated that the pathogenic fungi responsible for *Coix lacryma-jobi* L. infections that causing smut disease may also include members of the *Sporisorium* group or other physiological races. Therefore, given the limited number of strains reported in previous studies and the absence of systematic analysis, this study aims to isolate and identify the pathogenic fungi associated with *Coix lacryma-jobi* L. to elucidate their growth characteristics, nutritional and taxonomic status. This research could establish a foundation for understanding the subsequent stages of the infection mechanism of adlay smut and lay the groundwork for future advancements in the applications of *Ustilago coicis*.

## 2 Materials and methods

### 2.1 Plants

Samples were collected from naturally infected adlay leaves and inflorescences, which were gathered in 2022 at MuJia, Xingyi City, Guizhou Province (N 25.1158333 E 104.8683333). The materials were selected from the entire young fruit of adlay smut that were susceptible but not damaged, and stored at clean container for analysis.

### 2.2 Fungal isolation

Teliospores was weighted for 0.1 g, transferred into a 2 mL centrifuge tube and centrifuged at 5,000 rpm for 1 min. The supernatant was removed and added with 2 mL of sterilized distilled water to create a mixture. Teliospore suspension (100 μL) was collected and placed on a glass slide. The slide was incubated in complete darkness incubator with a relative humidity of 80% and temperature of 26 °C for teliospores germination, growth, and development. Additionally, intact and susceptible coix involucrum was selected and surface disinfected, and then teliospores were inoculated into Potato Dextrose Agar (PDA) solid medium which constituted with potato (200 g/L), agar powder (15 g/L), and glucose (20 g). Culture-specific colonies were incubated at approximately 26 °C for 10 days without contamination before being transferred to a new PDA solid medium through repeated processes (3 times) to obtain high-purity colonies.

### 2.3 Morphological identification

The bent and deformed stems and leaves of adlay smut with involucre were selected for analysis. Pathogenic fungi were isolated, purified, and identified. The morphology of the colony, teliospores, and mycelium of adlay smut at different stages were observed and analyzed. Additionally, the surface structure of teliospores, as well as mycelia and yeast-like sporidia were observed using scanning electron microscope (SEM) after being cultured in PDA medium for approximately 10 days. Samples of *Ustilaginaceae* mycelia and teliospore sori (1 mm × 2 mm) were fixed with fixative solution for electron microscopy immediately. After a 2 h fixation at room temperature, the samples were rinsed 3 times with a 0.1 M phosphate buffer solution (PBS, pH 7.4) for 15 min each time. A 1% osmic acid solution prepared by 0.1 M PBS (pH 7.4) was used to further fix the samples in darkness at room temperature for 2 h. Subsequently, another 3 rinses with 0.1 M PBS (pH 7.4) were conducted and lasting for 15 min each rinse. Samples were then immersed in ethanol solutions with concentrations of 30, 50, 70, 80, 90, 95, and 100% for 15 min each. They were subsequently dehydrated using isoamyl acetate for 15 min, dried with a critical point dryer, and then attached to conductive carbon film using double-sided adhesive tape. Afterward, an ion sputtering device was sprayed on gold for about 30 s. Finally, the specimens were observed under a SEM, and images can be captured accordingly.

### 2.4 Molecular identification

Purified mycelium was weighed for 0.1 g and total DNA was extracted by following the instructions of Ezup column-type fungal genomic DNA extraction kit. Primers were designed based on the flanking sequences of internal transcribed spacer (ITS), large subunit ribosomal DNA (LSU), and ATP synthase subunit 6 (ATP6) regions for PCR amplification (White et al., 1990; Stoll et al., 2005; Meng et al., 2021). The sequences were amplified from the DNA using the generic fungal primers ITS1 5′-TCCGTAGGTGAACCTGCGG-3′, ITS4 5′-TCCTCCGCTTATTGATATGC-3′, NL1 GCATATCAATAAGC GGAGGAAAAG, NL4GGTCCGTGTTTCAAGACGG, ATP6-F GGWGCTAGGAATCCTGMAAG, ATP6-R CTCTAAAYCTACCW GTACTTGG. PCR amplification products were conducted using 1% agarose gel electrophoresis. Sequence determination of the recombinant plasmid was performed by Sangon Bioengineering (Shanghai) Co., Ltd. in both directions.

Fungal sequences were imported into NCBI for a BLAST search and comparison. The sequences with high homology were analyzed, and the literature was consulted to download the homologous sequences. Double-stranded sequences for the ITS, LSU, and ATP6 were aligned and edited using Bioedit Sequence Alignment Editor. All sequences and alignments have been deposited in GenBank with the following accessions: OQ283646-OQ283704–OQ417285 and OQ283647–OQ283705–OQ417286, respectively (Supplementary Table 1). The ITS, LSU, and ATP6 sequences were imported into Sequence Matrix for multi-gene alignment, and the aligned sequences were constructed using PAUP* 4.0 b10. Phylogenetic trees were constructed using the maximum parsimony (MP) method. In the maximum parsimony (MP) analyses, trees were inferred using the heuristic search option with tree bisection and reconnection (TBR) branch swapping and 1,000 random sequence additions. Gaps were treated as missing data, and characters were given equal weight. Bootstrap analyzes were based on 1,000 replications, each with 10 replicates of randomly adding taxa in a stepwise manner.

### 2.5 Effect of different carbon and nitrogen sources on the growth of the pathogen

2 g of peptone, 1.0 g of potassium dihydrogen phosphate, 1.0 g of magnesium sulfate, 0.1 g of calcium chloride, 0.1 mg of vitamin B1 (VB1), and 0.1 mg of vitamin B6 (VB6) to 1,000 mL of distilled water. Glucose, sucrose, maltose, lactose, and mannose were each added as sole carbon sources at equal concentrations of 20 g/L. After sterilization, the various culture media were poured into Petri dishes and set aside. Under sterile conditions on a clean bench, a fungal cake was punched out using a sterile 6 mm diameter punch and placed in the center of each Petri dish. Each treatment was replicated three times, labeled, and incubated at 26 °C. Colony growth was observed after 14 days, and colony diameters were measured using the “crossover” method. This study investigated the effects of different carbon sources on the growth of *Ustilago coicis*. For nitrogen source experiments, a basal medium containing 20.0 g of glucose, 1.0 g of potassium dihydrogen phosphate, 1.0 g of magnesium sulfate, 0.1 g of calcium chloride, 0.1 mg of VB1, 0.1 mg of VB6, and 15 g of agar was prepared in 1000 mL of distilled water. Different nitrogen sources, peptone, beef extract, urea, potassium nitrate (KNO_3_), and ammonium sulfate ((NH4)_2_SO_4_) were each added at a concentration of 2%. The “crossover” method were employed to assess the effects of these nitrogen sources on the growth of *Ustilago coicis*.

### 2.6 Effect of different temperatures, pH levels, light conditions, and phytoexudates on the growth of the pathogen

Optimal temperature test: Prepare a basal medium by adding 2 g of peptone, 20.0 g of glucose, 1.0 g of potassium dihydrogen phosphate, 1.0 g of magnesium sulfate, 0.1 g of calcium chloride, 0.1 mg of vitamin B1, 0.1 mg of vitamin B6, and 15 g of agar to 1,000 mL of distilled water. Distribute the medium into six treatments at 18 °C, 22 °C, 26 °C, 30 °C, 33 °C, and 36 °C, with three biological replicates per treatment. Label the samples and place them in a constant-temperature incubator, incubating in the dark for 14 days. Measure colony diameters using the “crossover” method. Optimal pH test: Use the same basal medium as in the optimal temperature test. Adjust the pH of the medium to 5, 6, 7, 8, and 9 using 1 mol/L HCl and 1 mol/L NaOH, creating five treatments with three biological replicates each. Optimal Light exposure test: Using the basal medium described above, inoculate with the fungus and incubate under two light conditions: a 12-h light/12-h dark cycle and continuous darkness for 24 h, with three biological replicates per treatment. Best plant extract test: Using the basal medium described above, add plant extracts from potato, cornmeal, coix seed, wheat bran, and oat (each at a concentration of 20.0 g). Each treatment has three replicates. Incubate at 26 °C for 14 days, then measure colony diameters using the “crossover” method.

### 2.7 Nutrients of *Ustilago coicis*

Two *Ustilago coicis* fungal cakes with 6 mm in diameter each were inoculated into 100 mL of Potato Dextrose Broth (PDB) culture medium and incubated at a temperature of 26 °C with agitation at a speed of 140 rpm for 5 days. Subsequently, the mycelia were centrifuged and the resulting mycelial biomass was utilized for the determination of relevant nutrients. Each treatment was biologically replicated three times. Crude protein content was determined using the Kjeldahl method, total starch was quantified using an acid solution-DNS assay, crude fiber content was assessed through Van Soest method, polysaccharides were measured via phenol-sulfuric acid colorimetric approach, trace elements were analyzed by inductively coupled plasma-optical emission spectroscopy (ICP-OES/MS), and amino acids and vitamins were determined utilizing high performance liquid chromatography (HPLC).

### 2.8 Genome sequencing and assembly

Genomic DNA was extracted from two fungal strains using the E.Z.N.A.^®^ Fungal DNA Kit (OMEGA). The quality of the extracted DNA was assessed by measuring the 260/280 nm absorbance ratio, which was approximately 1.8. The DNA concentration met the requirements for subsequent experiments. The extracted DNA showed no obvious degradation, and agarose gel electrophoresis revealed clear bands without significant smearing. Starting with 1 μg of DNA, libraries were constructed using the TruSeq^™^ Nano DNA Sample Prep Kit to create short fragment libraries of 300–500 bp. The process included end repair, 3′ adenylation, and ligation of index adapters. Paired-end 2 × 150 bp sequencing was performed on the Illumina NovaSeq platform. The third-generation sequencing workflow followed the standard protocol provided by PacBio, including sample quality assessment, library construction, library quality control, and sequencing. The main steps were as follows: after obtaining genomic DNA (gDNA), the DNA was fragmented to an appropriate size using either G-tube tubes or the Megaruptor System. Subsequently, single-stranded overhangs were removed, and damage repair and end repair were performed to generate intact double-stranded insert fragments. Next, adapters were ligated to the double-stranded DNA to create SMRTbell libraries, resulting in circular templates. After adapter ligation, the ligation products were purified, and enzymatic treatment was applied to digest linear or internally damaged circular DNA molecules. Following enzyme treatment, size selection and recovery of the target library size range were performed using BluePippin or the Sage ELF System. Once library construction was complete, HiFi sequencing was carried out on the PacBio Sequel II platform. First, the software MaSuRCA v4.0.8^1^ was used to perform hybrid assembly of second-generation Illumina reads and third-generation long reads. Next, the software Canu v2.2^2^ was used to assemble the third-generation long reads. Then, the software MUMmer v4.0^3^ was employed to integrate the assemblies from MaSuRCA and Canu and to remove redundancies. The integrated genome sequence was polished with three rounds using the software Racon v1.4.20,^4^ followed by at least three rounds of base correction using the software Pilon v1.22^5^, ensuring approximately 99.9% accuracy of the assembled genome sequence.

### 2.9 Gene prediction and functional annotation

Using the reference genome for training, denovo gene prediction was performed with AUGUSTUS v3.2.3 software.^6^ Homology-based prediction was conducted using protein sequences from the reference genome: protein sequences were first rapidly aligned to the sample genome sequence, poor alignments were filtered out, and redundancies were removed. Then, Genewise v2.4.1^7^ was run for precise alignment to determine gene coding regions and intron regions. Finally, the gene sets obtained above were integrated using EVidence Modeler v1.1.1^8^ software to generate the protein-coding genes of the sample genome. rRNA was predicted using the RNAmmer v1.2 software,^9^ tRNA regions and tRNA secondary structures were predicted using the tRNAscan v2.0.7 software,^10^ and sRNA, snRNA, and miRNA were predicted using the Rfam v2.2 software.^11^ Dispersed repeat sequences were identified using Repeat Masker 4.1.6 software,^12^ and tandem repeat sequences in DNA were searched using the TRF v4.09 software.^13^ Repeat Masker searches for interspersed repetitive sequences by comparing the sequences against the known repeat sequence database Repbase. TRF simulates tandem repeat sequences by validating percentages and the frequency of adjacent pattern copy InDels, and uses statistical criteria to identify tandem repeats. Based on the genome assembly results, the predicted gene protein sequences were compared against the NR, eggNOG, KEGG, and GO databases using blastp (BLAST + 2.7.1, alignment criteria: *E*-value ≤ 1 × 10^−5^) to obtain annotation information for the protein-coding genes.

### 2.10 Gene family analysis and phylogenetic clustering

Comparative genomic analysis was conducted between the genomes of the two isolated fungal strains and the genomes of eight other smut fungi from the NCBI database (Table 1). The amino acid sequences of the 10 species were aligned and clustered into homologous gene families using OrthoMCL v2.0.3 software.^14^ Homologous genes that were present in single copies in all species were selected for multiple sequence alignment using MUSCLE v3.8.31 software.^15^ Model parameters were estimated using jModeltest v2.1.10 software,^16^ and a maximum likelihood phylogenetic tree was constructed using PhyML v3.0.^17^ Obtain the fossil divergence times of species from the website,^18^ estimate the divergence times using PAML MCMCtree v4.4,^19^ and incorporate the species’ fossil divergence times into the phylogenetic tree. Based on the gene family and divergence time tree results, we simulated a random gene birth and death rate, *λ*, using CAFE v1.6 software to predict the evolution of gene families in different species within each clade.

**Table 1:** Information of different smut fungus genomes.

BioProjects	Scientific name	Host
PRJEB7751	Bromivora smut	*Brachypodiu*
PRJNA14007	Maydis smut	*Zea mays* L.
PRJNA727466	Barley smut	*Hordeum vulgare*
PRJEB19311	Reilianum smut	*Sorghum*
PRJNA275631	Scitamineum smut	*Saccharum* sp
PRJNA554127	Graminicola smut	*Meadow smut*
PRJNA316802	Trichophora smut	*Echinochloa colona*
PRJNA793722	Adlay smut	*Coix lacryma-jobi* L.

### 2.11 Statistical analysis

The experimental data was organized using Excel 2010 and subjected to statistical analysis using SPSS 26.0. An independent sample *t*-test was employed to analyze significant differences between the two samples. Graphpad Prism 8.0 software was employed for data visualization and plotting purposes. The systematic evolutionary tree was constructed utilizing PAUP 4.

## 3 Results

### 3.1 Morphological characteristics of teliospore balls

**Figure 1:**
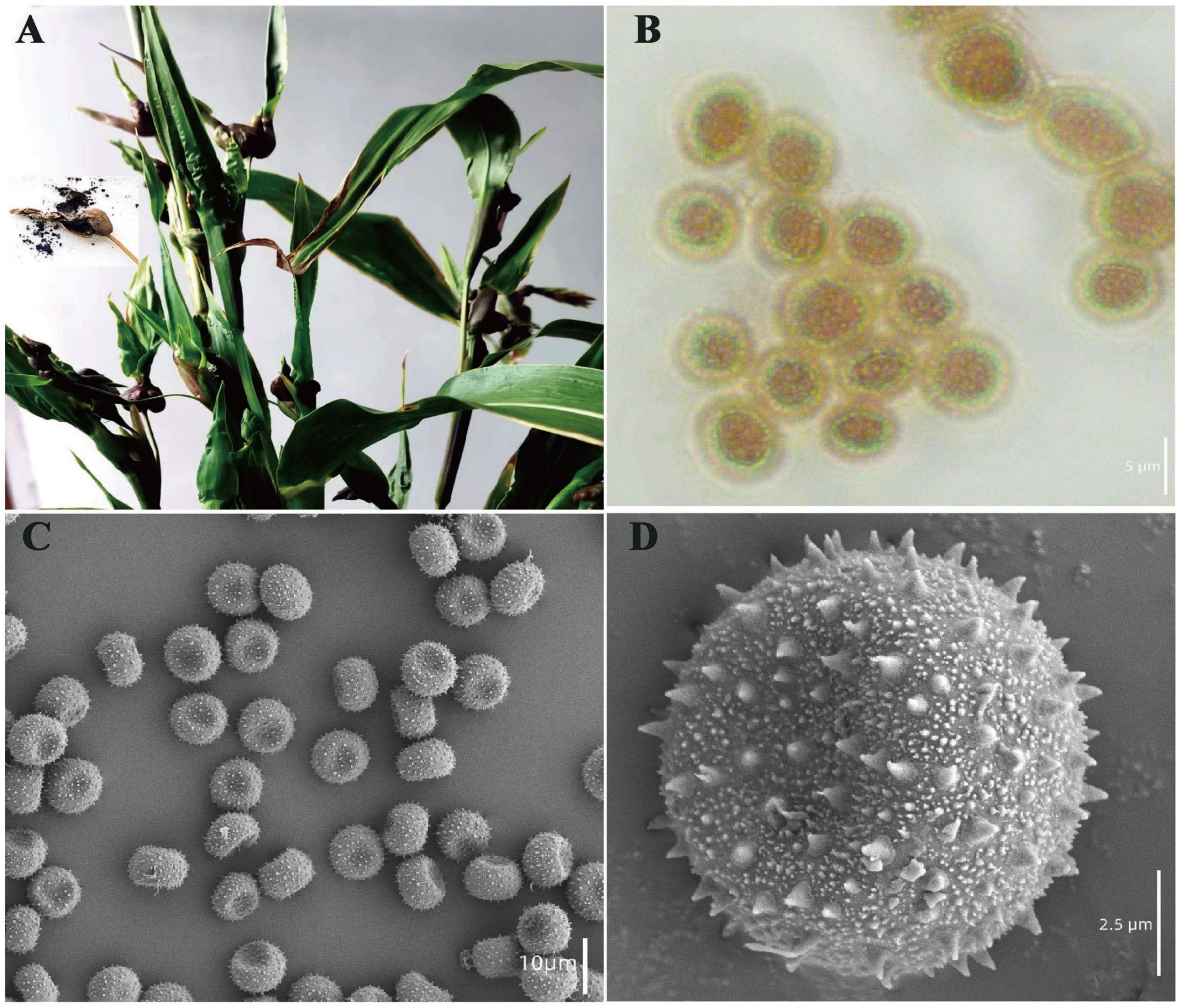
Morphology of teliospores of adlay smut. **(A)** Involucre of adlay smut, **(B)** teliospores morphology under microscope, **(C,D)** SEMs under different magnifications.

The occurrence of adlay smut is attributed to basidiomycete fungi, which caused a systemic disease. The primary symptom is the development of sori in host’s fructification, stems, and leaves, forming an enlarged ovary which is involucre (Figure 1A). The dark sori were the dormant teliospores of adlay smut which appeared spherical or oval under microscope with the size range of 4.04–5.05 μm × 7.14–7.97 μm. The teliospores showed the color of light yellow with spines (Figure 1B). It was wheel-shaped with slightly concave ends and the spikes were clearly visible under the SEM (Figures 1C,D).

### 3.2 Morphology of isolated strains

**Figure 2:**
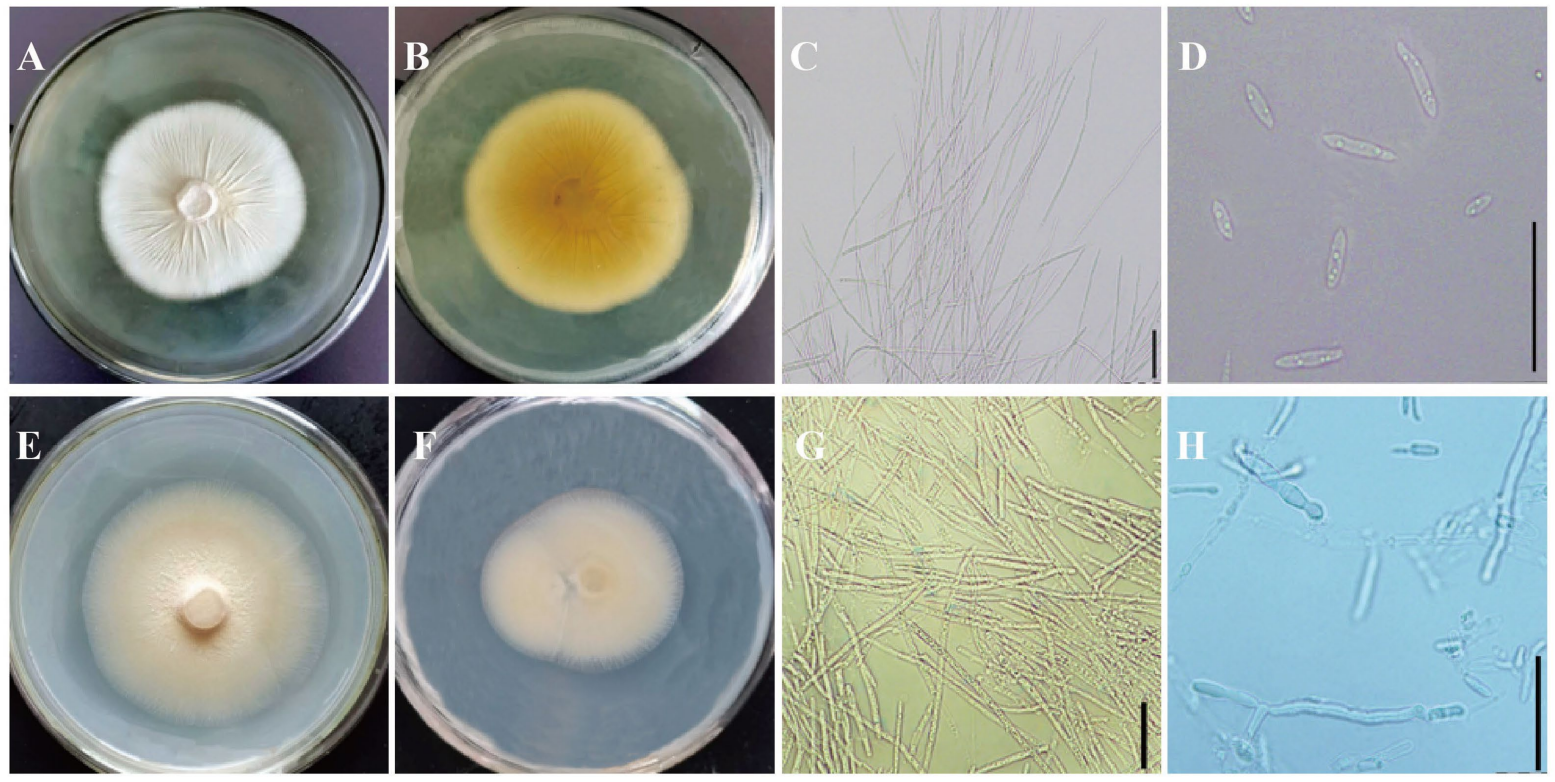
Morphology of *Ustilago coicis*. **(A,B)** The front and back sides of the ZC 202104 colony plate, respectively, **(C)** mycelium of ZC 202104, **(D)** basidiospores of ZC 202104, **(E,F)** the front and back sides of the ZC202205 colony plate, respectively, **(G)** mycelium of ZC 202205, **(H)** basidiospores of ZC 202205.

**Figure 3:**
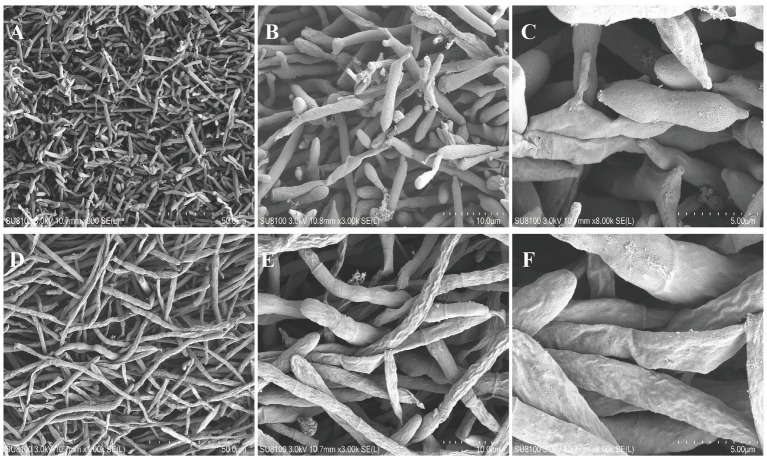
Scanning electron micrographs (SEMs) of *Ustilago coicis*. **(A–C)** The mycelium morphology and basidiospores of ZC 202104 under different magnifications under the SEM. **(D–F)** The mycelium morphology and basidiospores of ZC 202205 under different magnifications under the SEM. Arrows point to basidiospores.

Two strains with different colony morphologies were isolated from the involucre of adlay smut which were labeled as ZC 202104 and ZC 202205, respectively. They grew slowly on PDA medium taking approximately 20 days to cover the plate. The colonies of ZC 202104 appeared white with yeast-like folds in the center with smooth opaque edges (Figures 2A,B). The spores were less branched and fusiform shaped with a distinct nucleoplasm, measuring 1.32–2.17 μm × 2.86–9.5 μm in size (Figures 2C,D). The mycelium was observed under the electron microscope after being cultured for 10 days, which showed that the asidiospores were produced at the terminal ends of branches and mycelium was deformed. Distinct diaphragms were observed at the sporulation site, accompanied by numerous basidiospores exhibiting a gourd-shaped morphology with a rough surface and a cotton-like fluff (Figures 3A–C). The colony of ZC 202205 appeared off-white with a central fluff and lacking yeast-like folds accompanied with smooth and translucent edges (Figures 2E,F). The mycelium of ZC202205 was thicker and shorter than that of ZC 202104 with fewer branches. Basidiospores were connected by 2–3 ellipses with a size range of 1.41–2.39 μm × 3.16–9.47 μm (Figures 2G,H). Under electron microscopy, basidiospores were rarely observed while the surface of mycelium appeared more shrunken with a cotton-like fluff and shaped like large intestinal nodules (Figures 3D–F).

### 3.3 Dynamic development process of teliospores of adlay smut

**Figure 4:**
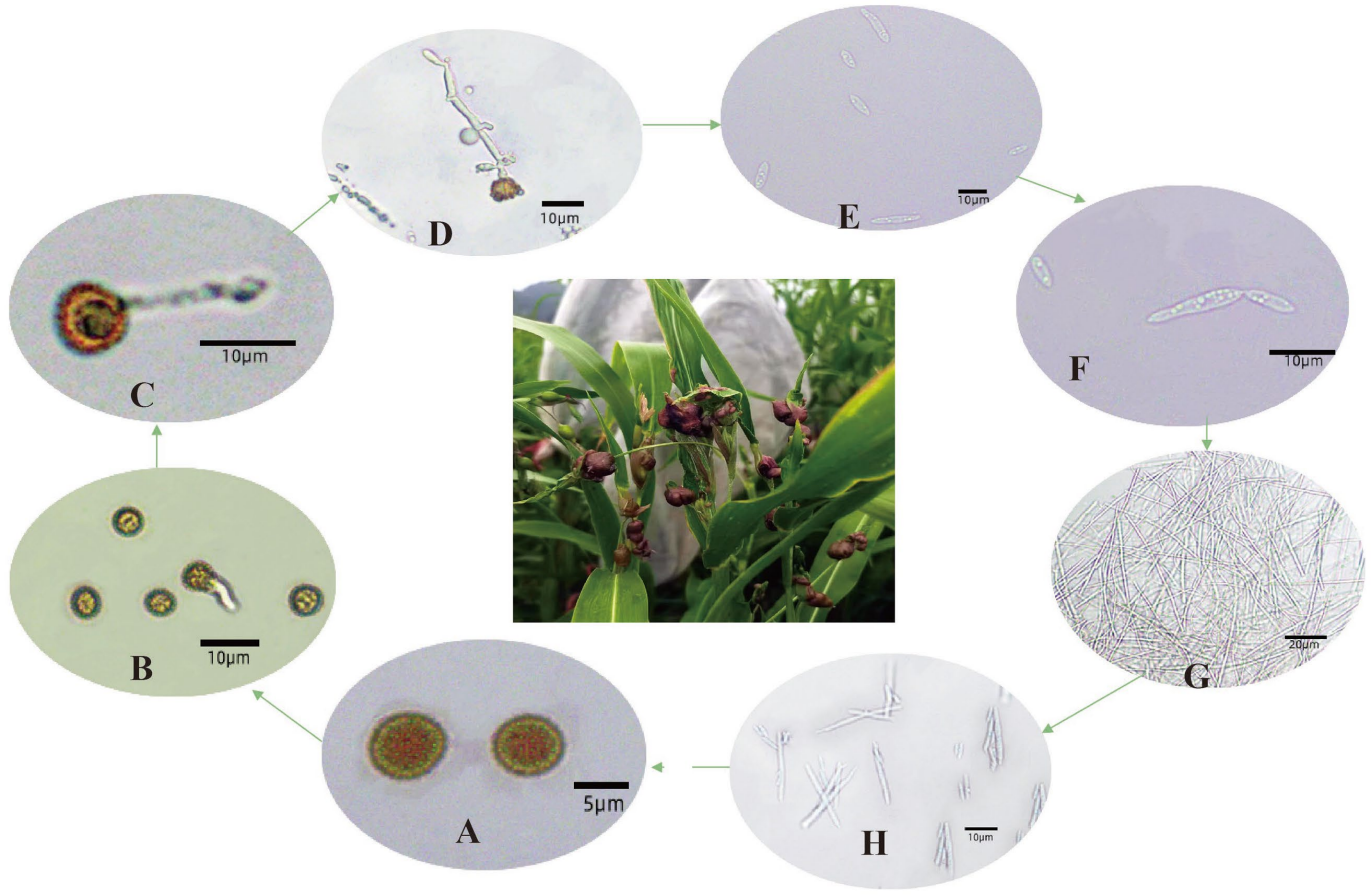
Teliospore development of adlay smut. **(A)** Teliospore, **(B)** germination of teliospore, **(C)** shrinkage of teliospore, **(D)** promycelium produces basidiospores, **(E)** basidiospores, **(F)** saprophytic growth, **(G)** mycelium, **(H)** mycelium produces basidiospores(14 days).

The subphylum *Ustilaginomycotina* is usually dimorphic producing a saprobic haploid yeast phase and a parasitic dikaryotic hyphal phase (Castanheira and Pérez-Martín, 2015). In this study, microscopy observations revealed that teliospores germinate within approximately 2 days, giving rise to premycelium (Figures 4A,B). When the premycelium reached about 8 μm in size, the teliospores began to shrink and deform, while the premycelium continued to grow (Figure 4C). After 10 days, distinct diaphragms can be observed on the mycelium, with basidiospores being produced at both the top and side ends of the premycelium (Figure 4D). These basidiospores continued to develop into slender mycelium after detaching from the premycelium (Figures 4E–G). Basidiospores were rarely observed during the first 7 days. Accompanied by mycelium deformation and fragmentation, basidiospores were produced on the sides of the mycelium at day 14 (Figure 4H). At day 20, elongated mycelia were absent, and short fusiform mycelia were observed along with a large number of basidiospores (Figure 4).

### 3.4 Phylogenetic tree analysis

A phylogenetic tree was constructed based on multi-gene sequences from the internal transcribed spacer (ITS), large subunit ribosomal DNA (LSU), and ATP synthase subunit 6 (ATP6) regions. Phylogenetic analyzes of each locus were conducted under MP and ML criteria using *Moesziomyces bullatus* as the outgroup (Stoll et al., 2005; Zhang et al., 2013). After cluster analysis, it was determined that the target strains ZC 202104 and ZC 202205 were grouped together with *Ustilago coicis* on the main branch. The phylogenetic tree in this study were consistent with the results Zhang et al. (2013), indicating the reliability of the constructed phylogenetic tree. Combined with the results of morphological identification, it can be inferred that the strains we isolated belong to *Ustilago* family. Furthermore, the target strains ZC 202104 and ZC 202205 were found to be clustered together with 88% bootstrap support (Supplementary Figure 1), confirming that they belong to the species *Ustilago coicis*. However, they were identified as two distinct physiological races.

### 3.5 Effect of different carbon and nitrogen sources on the growth of *Ustilago coicis*

**Figure 5:**
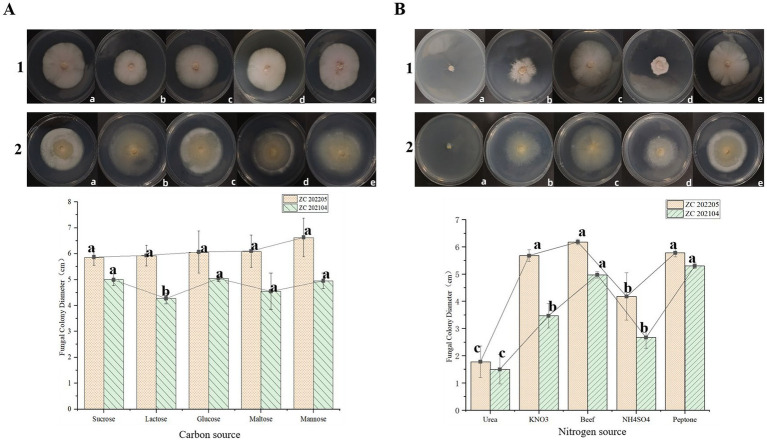
Effect of different carbon and nitrogen sources on the growth of *Ustilago coicis*. **(A)** Effect of different carbon sources on the growth of *Ustilago coicis*. **(B)** Effect of different nitrogen sources on the growth of *Ustilago coicis*. 1 and 2 are images of strains ZC202104 and ZC202205 growing on petri dishes, respectively. In **(A)**, images **A–E** for 1 and 2 correspond to *Ustilago coicis* grown with sucrose, lactose, glucose, maltose, and mannose, respectively. In **(B)**, images **A–E** for 1 and 2 correspond to *Ustilago coicis* grown with urea, KNO_3_, beef extract, NH_4_SO_4_, and peptone, respectively.

After 14 days of incubation, the mycelial growth of ZC 202104 was the fastest, and its colony diameter was the largest (51.0 mm) when glucose was used as the carbon source, however, its growth was slowest, and colony diameter was the smallest (42.7 mm) when lactose was used as carbon source. There was a significant difference between the colony diameters of ZC 202104 growing on glucose and lactose, and the colony diameters of those growing on other carbon sources were between the two. In ZC 202205, mannose had the largest colony diameter (66.3 mm), while sucrase had the smallest colony diameter (58.7 mm), and there was no significant difference between the carbon sources (Figure 5A). Furthermore, the mycelial growth of ZC202104 was the fastest, and its colony diameter was the largest (53.0 mm) when peptone was used as the nitrogen source, while the utilization effect of urea was the lowest, resulting in the smallest colony diameter (15.0 mm). There was a significant difference between the colony diameters of ZC 202104 growing on peptone and urea. The colony diameters of *Ustilago coicis* growing on other nitrogen sources were between the two, In ZC 202205, the colony diameter of beef paste was the largest (61.8 mm), while that of urea was the smallest (13.3 mm). There was a significant difference between the colony diameters of ZC 202205 growing on beef paste and urea, and the colony diameters of those growing on other carbon sources were between the two. And there are differences in the morphology of the two strains growing on different nitrogen sources (Figure 5B). This research result indicates that ZC202104 and ZC202205 strains not only differ in morphology, but also in the types of carbon and nitrogen sources they utilize.

### 3.6 Effect of different temperature, pH levels, light, and plant extracts

**Figure 6:**
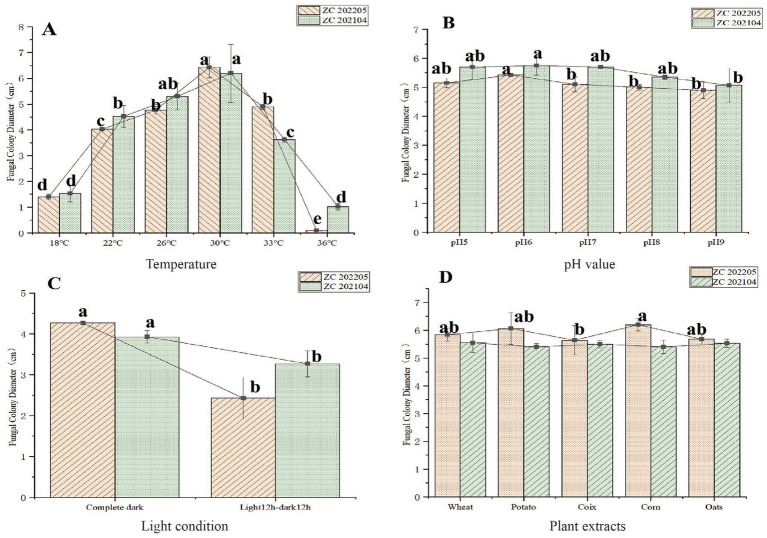
The influence of different growth conditions on *Ustilago coicis*. **(A)** Effect of different temperatures, **(B)** pH levels, **(C)** light conditions, **(D)** phytoexudates.

After 14 days of culture, average radial hyphal growth was measured at each temperature, and the results are shown in the Figure 6A. At 30 °C, strains ZC202104 and ZC 202205 exhibited the highest radial hyphal growth, reaching 62.0 mm and 64.3 mm, respectively. At 18 °C and 36 °C, both strains showed little growth, and colony diameters varied significantly at different temperatures. These results indicate that *Ustilago coicis* is a thermophilic fungus compared to other common fungi. At pH 6, the maximum radial mycelial growth of ZC202104 and ZC 202205 was 57.5 mm and 54.3 mm, respectively. At pH 9, the minimum radial mycelial growth was 50.7 mm and 49.0 mm, respectively. Mycelial growth rates at other pH levels were intermediate (Figure 6B). These results indicate that ZC202104 and ZC 202205 strains are equally sensitive to pH, with an optimum pH of 6. However, ZC202104 and ZC 202205 grew more slowly under 12 h light/12 h dark conditions, resulting in smaller colony diameters of 32.7 mm and 24.3 mm, respectively. Under continuous darkness, ZC202104 and ZC 202205 strains grew more rapidly, reaching colony diameters of 39.3 mm and 42.4 mm, respectively. The differences in colony diameters were significant, indicating that full darkness was more conducive to mycelial growth (Figure 6C). Plant extracts are often rich in nutrients. In this study, 2 g of peptone, 1.0 g of potassium dihydrogen phosphate, 1.0 g of magnesium sulfate, 0.1 g of calcium chloride, 0.1 mg of VB1, and 0.1 mg of VB6 were added to 1,000 mL of distilled water as a basal culture medium. Different plant extracts were added. As shown, the colony diameter of ZC202104 was the largest, at 55.5 mm, in the culture medium supplemented with wheat bran. The colony diameter was the smallest, at 53 mm, in the culture medium supplemented with corn. There were no significant differences between the plant extracts. The colony diameter of ZC202205 was the largest, at 62.0 mm, in the culture medium supplemented with corn. The colony diameter was the smallest, at 56.4 mm, in the culture medium supplemented with Coix. Significant differences were observed between the plantDextracts (Figure 6D).

### 3.7 Nutrients of *Ustilago coicis*

The contents of crude protein, total starch, crude fiber, and polysaccharide in both *Ustilago coicis* strains were presented in Table 2. Among these components, the crude protein, crude fiber, and polysaccharide content of ZC 202104 were 11.98 ± 1.71 mg/g, 0.65 ± 0.13 mg/g, and 5.84 ± 0.26 mg/g, respectively, which exhibited higher levels compared to ZC 202205. However, ZC 202205 contained slightly higher total starch content than ZC 202104. The contents of mineral elements including potassium (K), sodium (Na), calcium (Ca), magnesium (Mg), and iron (Fe) in strains of *Ustilago coicis* were listed in Table 2. Notably high levels of Ca were observed in ZC 202104 (0.57 mg/g) and ZC 202150 (0.52 mg/g). Additionally, in ZC 202104, the contents of K, Na, Mg and Fe were measured as 0.27 ± 0.03, 0.44 ± 0.09, 0.32 ± 0.10, and 39.31 ± 5.54 mg/kg, respectively. Whereas in ZC 202205, the contents of K, Na, Mg and Fe were 0.25 ± 0.03, 0.29 ± 0.06, 0.29 ± 0.08, 41.96 ± 4.19 mg/kg, respectively. Therefore, *Ustilago coicis* stands out as a fungus abundant in diverse minerals. The presence of vitamins, including VA, VB1, VB2, VB3, and VC in *Ustilago coicis* were shown in Table 2. Among these measured vitamins, VA exhibited the highest concentration with values of 1740.22 ng/g and 1563.31 ng/g for ZC 202104 and ZC 202205, respectively, followed by VC with concentrations of 184.72 ng/g and 176.49 ng/g for ZC 202104 and ZC 202205, respectively. Seven B-vitamins were evaluated in two strains and the concentrations of VB1, VB2, and VB3 were relatively high. Specifically, in strain ZC 202104, the values for VB1, VB2, and VB3 were measured at 5.71 ± 0.05, 3.81 ± 0.01, and 3.42 ± 0.01 ng/g, respectively. Whereas in strain ZC 202205, these values were recorded as 4.62 ± 0.04 ng/g for VB1, 5.42 ± 0.01 ng/g for VB2 and 0.78 ± 0.00 ng/g for VB3.

**Table 2:** Main nutrients, inorganic elements and vitamin contents of *Ustilago coicis* (mean ± RSD, *n* = 3).

Name	ZC 202104	ZC 202205
Crude protein (mg/g)	11.98 ± 1.71	8.17 ± 0.85
Total starch (mg/g)	30.06 ± 2.54	32.41 ± 2.81
Crude fiber (%)	0.65 ± 0.13	0.24 ± 0.04
Polysaccharide (mg/g)	5.84 ± 0.26	3.74 ± 0.34
K (mg/g)	0.27 ± 0.03	0.25 ± 0.03
Na (mg/g)	0.44 ± 0.09	0.29 ± 0.06
Ca (mg/g)	0.57 ± 0.11	0.52 ± 0.14
Mg (mg/g)	0.32 ± 0.10	0.29 ± 0.08
Fe (mg/kg)	39.31 ± 5.54	41.96 ± 4.19
VA (ng/g)	1740.22 ± 10.92	1563.31 ± 25.13
VC (ng/g)	184.72 ± 0.19	176.49 ± 0.37
VB1 (ng/g)	5.71 ± 0.05	4.62 ± 0.04
VB2 (ng/g)	3.81 ± 0.01	5.42 ± 0.01
VB3 (ng/g)	3.42 ± 0.01	0.78 ± 0.00
VB5 (ng/g)	0.17 ± 0.00	0.11 ± 0.00
VB6 (ng/g)	0.11 ± 0.00	0.13 ± 0.00
VB9 (ng/g)	0.11 ± 0.00	0.09 ± 0.00
VB12 (ng/g)	0.02 ± 0.00	0.05 ± 0.00

**Figure 7:**
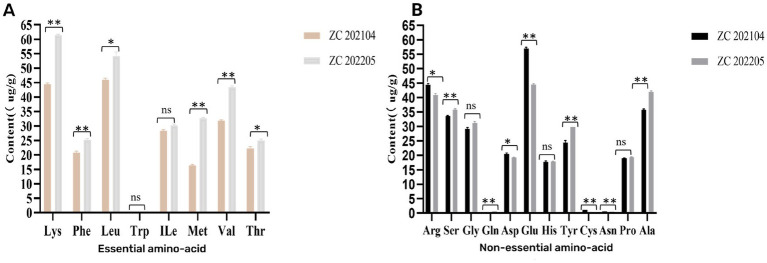
Amino acid content of *Ustilago coicis*. **(A)** Essential amino acid; **(B)** non-essential amino acid. (ns, no significant difference; ^*^p < 0.05 and ^**^p < 0.01 (Student *t*-test)).

Eight essential amino acids including lysine, tryptophan, phenylalanine, methionine, threonine, isoleucine, leucine and valine were detected in two *Ustilago coicis*. Levels of essential amino acids in ZC 202104 and ZC 202205 were quantified as 210.22 μg/g and 272.24 μg/g, respectively. Moreover, ZC 202205 exhibited higher essential amino acids compared to ZC 202104, while the total amino acid contents were measured as 493.74 μg/g and 554.74 μg/g, respectively. The ratio of essential amino acids to total amino acids (EAA/TAA) in ZC 202104 and ZC 202205 were 42.58 and 49.08%, respectively. The analysis of the amino acid composition revealed that glutamic acid exhibited the highest concentration in ZC202104 with a value of 56.99 μg/g, followed by leucine at a concentration of 46 μg/g. Additionally, lysine displayed the highest content of 61.43 μg/g in ZC202205, followed by leucine with the level of 54.16 μg/g (Supplementary Table 2 and Figure 7). Based on these analysis results, *Ustilago coicis* can be considered as high-quality protein resource due to its high protein content.

### 3.8 Genome prediction and assembly status

The genome assemblies of two *Ustilago* strains, ZC202104 and ZC202205 (GenBank accession number: PRJNA1338800), both estimated at ~22 Mb in size. ZC202104 spans 20,160,870 bp with a GC content of 53.83%, while ZC202205 covers 20,267,296 bp with a GC content of 53.75%. Contiguity analysis revealed N50 values of 1,098,121 bp for ZC202104 and 876,989 bp for ZC202205. Gene prediction identified 5,873 coding transcripts in ZC202104, with an average length of 1,978 bp, compared to 5,869 transcripts averaging 1,983 bp in ZC202205. The cumulative length of all coding sequences reached 11,617,300 bp in ZC202104 and 11,638,980 bp in ZC202205. Functional annotation classified 45 transcripts as secretory proteins, 456 as intracellular proteins, and 246 as membrane proteins in ZC202104; for ZC202205, the corresponding counts were 44 secretory, 451 intracellular, and 249 membrane proteins. Non-coding RNA annotation detected 131 tRNAs, 40 rRNAs (2 18S, 3 28S, 35 5S), and 3 snRNAs in ZC202104. ZC202205 harbored a larger non-coding RNA repertoire, including 142 tRNAs, 52 rRNAs (8 18S, 9 28S, 35 5S), and 3 snRNAs. BUSCO assessment yielded a completeness score of 97.9% for both assemblies, confirming high-quality, near-complete genome representations (Table 3). Repeat element analysis identified 2,792 interspersed repeats in ZC202104, accounting for 1.0627% of the genome, compared to 2,736 elements in ZC202205 (1.0054% of genome coverage). These included long terminal repeats (LTRs), DNA transposons, long interspersed nuclear elements (LINEs), short interspersed nuclear elements (SINEs), rolling-circle (RC) elements, small cytoplasmic RNAs (scRNAs), and unclassified repeat types. Tandem repeat sequences were more abundant in ZC202205 (4,604 elements, 0.8694% genome coverage) than in ZC202104 (4,425 elements, 0.8238% genome coverage).

**Table 3:** Genome assembly, annotation and quality assessment in adlay smut fungus *Ustilago coicis*.

Category	Statistic	
	ZC202104	ZC202205
Assembly size (bp)	20,160,870	20,267,296
Scaffold N50 length (bp)	1,098,121	876,989
Scaffold N90 length (bp)	537,308	543,967
G + C (%)	53.83	53.75
Scaffolds	28	25
Longest scaffold (bp)	3,277,850	2,636,974
Shortest scaffold (bp)	1,604	28,429
Complete BUSCOs	742	742
Complete and single BUSCOs	741	739
Complete and duplicated	1	3
Fragmented BUSCOs	4	4
Missing BUSCOs	12	12
Complete and single-copy BUSCOs (S)%	97.8%	97.5%
Complete BUSCOs (C)%	97.9%	97.9%

### 3.9 Gene function annotation

The genomes of the two *Ustilago* strains were aligned and annotated using the NR, GO, KOG, and KEGG databases. ZC 202104 had 5,843 genes annotated in the NR database, accounting for 99.49% of the total annotated genes, while ZC 202205 had 5,840 genes annotated in the NR database, accounting for 99.51% of the total annotated genes. The NR database annotation results showed that the two strains had the highest number of genes aligned to *Ustilago trichophora*, accounting for 91.58 and 91.68%, respectively. When the protein-coding sequences of the ZC 202104 and ZC 202205 genomes were aligned to the GO database, they were primarily annotated to three GO categories: biological process, molecular function, and cellular component (Supplementary Figure 2). Within the biological process category, both ZC 202104 and ZC 202205 strains were most highly annotated in the cellular process category, with 2,737 and 2,738 genes, respectively. In terms of cellular components, the two strains had the most annotated genes in the cellular anatomical entity, with 2,731 and 2,734 genes, respectively. This may indicate that the cell membrane and nucleus play a significant role in environmental adaptation. Regarding molecular function, both strains had the most genes annotated to catalytic activity, with 1,383 genes each. Comparison and annotation of protein-coding sequences from the ZC 202104 and ZC 202205 genomes using the KEGG database revealed that the majority of genes in both strains were annotated to metabolic pathways (Supplementary Figures 3A,B). The KEGG database includes four major categories of protein function classification: information storage and processing, cellular processes and signaling, metabolism, and missing function descriptions. These four categories contain 25 subcategories. In ZC 202104, 531 genes have unknown functions. The largest number of genes, 356, are involved in posttranslational modification, protein turnover, and chaperones, followed by 307 genes involved in intracellular trafficking, secretion, and vesicular transport, and 301 genes involved in translation, ribosomal structure, and biogenesis. In ZC 202205, 529 genes have unknown functions. The largest number of genes, 356, are involved in posttranslational modification, protein turnover, and chaperones, followed by 311 genes involved in intracellular trafficking, secretion, and vesicular transport, and 302 genes involved in translation, ribosomal structure, and biogenesis. This may indicate that *Ustilago coicis* adapts to its environment and host plant through specialized protein synthesis and processing, which facilitates signal transduction. CAZY library annotation: Carbohydrate-active enzymes (CAZYmes) play a crucial role in the infection of host cells by plant pathogens. Fungi rely on these enzymes to break down plant cell walls for infection and also decompose and utilize carbon compounds and polysaccharides within the cell walls for fungal reproduction. CAZY enzymes primarily encompass six families: glycoside hydrolases (GHs), glycoside transferases (GTs), polysaccharide lyases (PLs), carbohydrate esterases (CEs), carbohydrate binding modules (CBMs), and accessory enzymes (AAs). ZC 202104 and ZC 202205, respectively, have a total of 227 and 229 genes annotated to the CAZY library, with GHs comprising the largest number of genes, at 107 and 109, respectively (Supplementary Figures 3C,D).

Although the gene function annotations of ZC 202104 and ZC 202205 are overall similar, there are significant differences in key enzymes, metabolic pathways, and functional characteristics. ZC 202104 possesses homogentisate 1,2-dioxygenase (HGD, hmgA), a key enzyme in tyrosine metabolism, which can specifically degrade homogentisate, enabling it to efficiently utilize tyrosine-containing substrates, a capability that is rare in most strains. It also has phenylacetate 2-hydroxylase (PHAA, PHACA, CYP504A1), which can hydroxylate phenylacetic aromatic compounds, giving it a clear survival advantage in environments containing aromatic pollutants and great bioremediation potential. Additionally, it contains fumarylacetoacetate hydrolase (FAH, fahA) and 2,3-dihydroxybenzoate decarboxylase (DHBD), which further complete the degradation pathway of complex aromatic substrates. In terms of survival strategies in oligotrophic environments, ZC 202104 possesses 3-deoxy-7-phosphoheptulonate synthase (E2.5.1.54, aroF, aroG, aroH), allowing it to synthesize phenylalanine, tyrosine, and tryptophan independently without relying on environmental supply, making it more competitive in nutrient-poor environments, which is consistent with the previously observed high content of amino acids in this strain. Additionally, it has hydroxymethylglutaryl-CoA lyase (HMCCL, hmgL), which participates in both branched-chain amino acid (valine, leucine, isoleucine) metabolism and butyrate metabolism, allowing it to use multiple non-carbohydrate substrates as energy sources.

ZC 202205, on the other hand, possesses ADE5 (K11788), a bifunctional enzyme that participates simultaneously in purine metabolism, global metabolism, and biosynthetic pathways, efficiently catalyzing key steps in purine synthesis to meet the nucleotide demands for rapid growth. It also has THI5 (K18278), participating in thiamine metabolism, global metabolism, and biosynthetic pathways, responsible for pyrimidine precursor synthesis, thereby achieving coordinated regulation of vitamin B1 and pyrimidine synthesis, consistent with the previous finding that this strain is rich in B vitamins. In terms of environmental adaptation, ZC 202205 contains the central regulatory factor RAPTOR (K07204), which is associated with autophagy, mTOR, PI3K-Akt, AMPK, and eight other key signaling pathways, allowing precise sensing of nutrient levels; it can proliferate rapidly when nutrients are abundant and initiate autophagy to maintain survival under nutrient scarcity, demonstrating strong environmental adaptability. It also possesses the zinc transporter SLC39A9 (ZIP9, K14715), which can accurately regulate intracellular zinc ion concentration, meeting enzyme activity demands while expelling excess zinc in high-zinc environments, matching the observation that in the main cultivation area of *Coix lacryma-jobi*, Qianxinan Prefecture, although soil heavy metal content is high, black powdery mildew incidence is severe. Moreover, its fumarate hydratase (fumC, FH) and adenosine kinase (ADK) participate in multiple metabolic pathways, enabling flexible switching between aerobic respiration, anaerobic fermentation, and mixotrophic modes, adapting to varying oxygen and nutrient conditions, providing broad-spectrum survival capability.

### 3.10 Screening and functional annotation of potential virulence-related genes and candidate effector proteins

The genome of this fungus was annotated and compared with the PHI database, resulting in five categories: reduced virulence, unaffected pathogenicity, loss of pathogenicity, lethal, and increased virulence. ZC202104 has a total of 198 genes, and Z202205 has a total of 199 genes. Among the five categories, the two strains have the same number of genes except for the category of loss of pathogenicity, where the gene counts differ (Supplementary Figure 4). The DFVF database^20^ provides classifications of plant diseases, such as invasive candidal disease and smut (e.g., corn smut), facilitating the exploration of disease mechanisms in the pathogen infecting adlay. We annotated the two strains, ZC202104 and Z202205, using the DFVF database, and the analysis results are shown in Supplementary Figure 4. In both strains, over 30% of the genes are associated with invasive candidal disease, and more than 10% are related to corn smut. These findings suggest that ZC202104 and ZC 202205 may share similar pathogenic mechanisms with “invasive candidal disease” and “Corn smut” in infecting adlay. Combined with previous gene function analysis, ZC202104 may degrade defensive phenolic substances within adlay through the aromatic metabolism pathway, thereby overcoming the host’s first line of immune defense, ZC202205, on the other hand, may rely on core regulatory factors such as RAPTOR to sense nutrient signals within *Coix lacryma-jobi* cells, regulating the balance between autophagy and proliferation to achieve intracellular colonization. These two may cooperate or act independently through different strategies to complete the infection process.

To accurately predict fungal effectors, we used EffectorP,^21^ a specialized tool designed to identify pathogen-secreted virulence factors that translocate into host cells or apoplastic spaces to manipulate immune responses and enable colonization. In strain ZC202104, we identified 1,407 cytoplasmic effectors, representing 23.96% of the total proteins, with 437 of these (confidence score ≥0.8) prioritized for subsequent functional validation and studies on pathogenic mechanisms. Additionally, we detected 77 apoplastic effectors (1.31%) and 4,339 non-effectors. Overall, approximately 26% of the proteins were classified as effectors (including cytoplasmic, apoplastic), indicating strong pathogenic potential. Strain ZC202205 exhibited a similar profile, with 1,413 cytoplasmic effectors (24.08%, 418 with confidence ≥ 0.8), 75 apoplastic effectors (1.28%), and 4,333 non-effectors. Approximately 26.17% of proteins were effectors, confirming a comparable high pathogenic potential to strain ZC202104 (Supplementary Table 3).

### 3.11 Gene family clustering and phylogenetic analysis of smut

**Figure 8:**
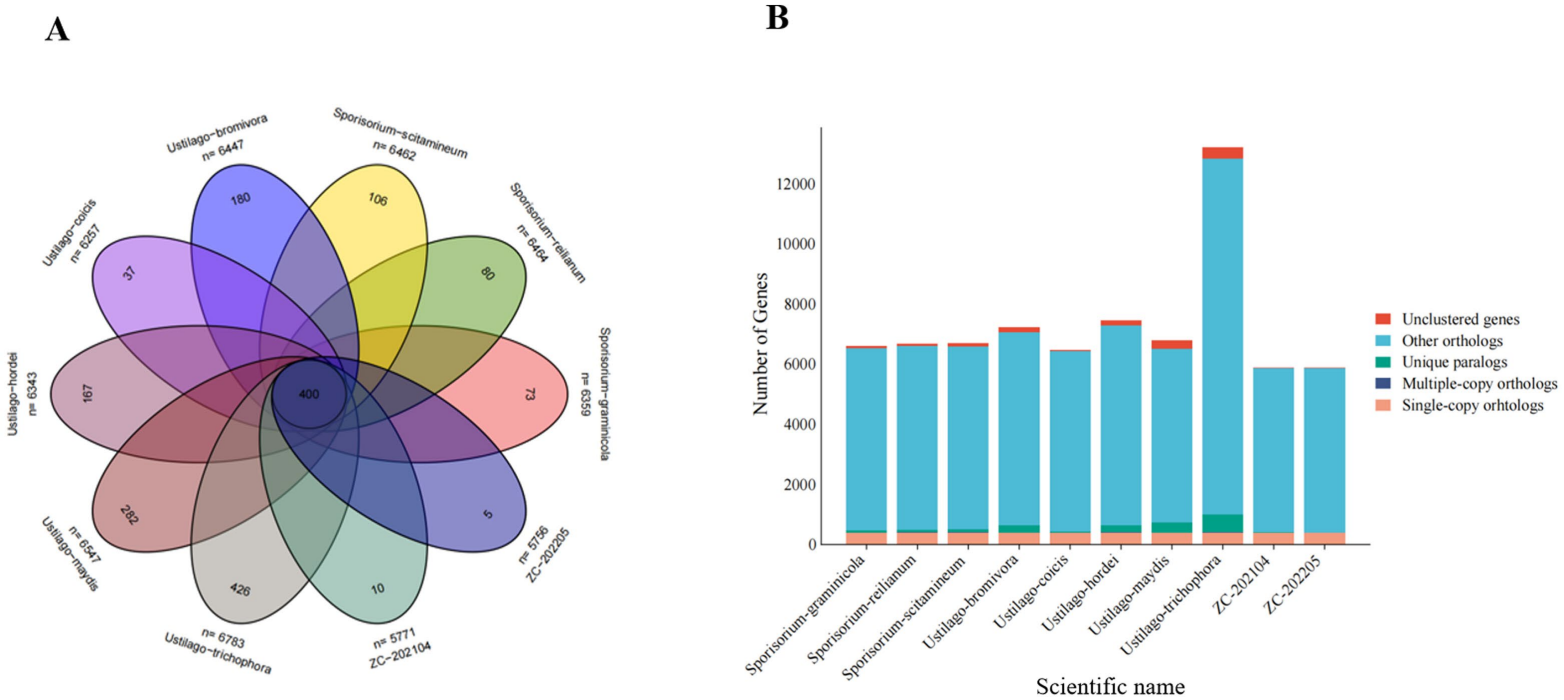
Statistics and clustering of homology genes in seven species of smut fungi: **(A)** Venn plot of homologygenes’ statistics and **(B)** bar chart of orthologs’ clustering. The middle circle represents all gene family number and the oval figures are unique genes, outside figures are species and their protein-coding numbers.

A gene family is a group of genes that share a common ancestor, different genes within a family often share similar structures and functions. The whole genome sequences of eight smut strains (PRJEB7751, PRJNA14007, PRJNA727466, PRJEB19311, PRJNA275631, PRJNA554127, PRJNA316802, and PRJNA793722) were used together with the genomes of strains ZC202104 and Z202205 to analyze predicted protein-encoding homologous gene families. Results showed that the 10 smut strains had between 5,756 and 6,783 protein-coding genes, including 400 shared gene families, while the number of unique genes for each of the 10 species ranged from 5 to 426. Regarding the number of genes in specific families, the genomes of strains ZC202104 and Z202205 both contained 400 single-copy genes and 4 multi-copy genes, including 10 and 6 specific genes, 5,449 and 5,455 other genes, and 10 and 6 unclassified genes, respectively (Figure 8).

**Figure 9:**
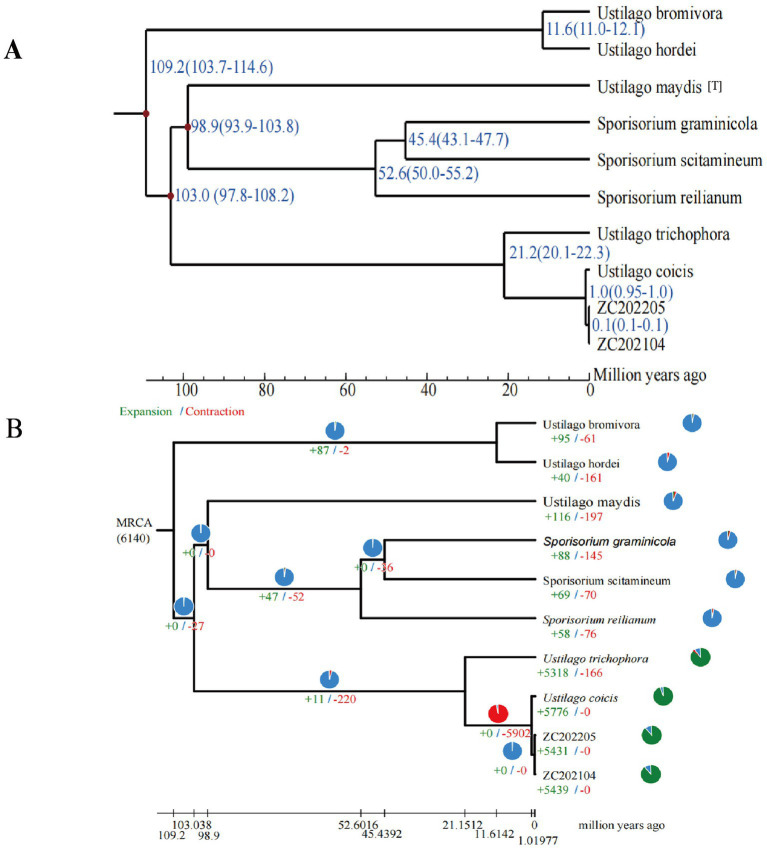
Divergence time tree. **(A)** Phylogenetic tree base on single copy genes; **(B)** phylogenetic tree base on gene families. Blue represents genes that have not changed, green and red correspond to expansion and contraction in the upper left corner.

**Figure 10:**
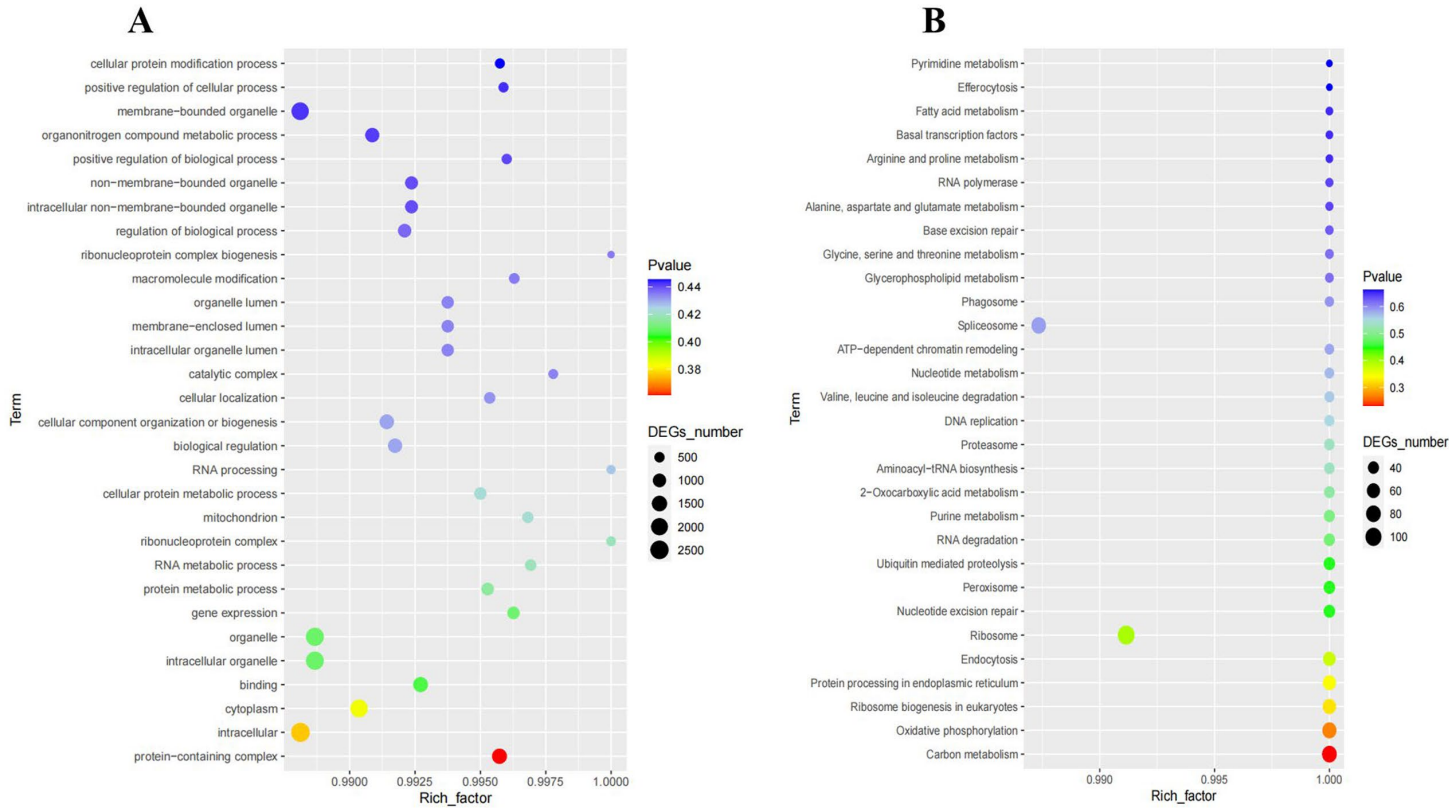
Enrichment results. **(A)** GO term enrichment functions; **(B)** KEGG pathway enrichment. The horizontal axis represents the enrichment factor, and the vertical axis represents the enriched functions. Larger circles indicate a relatively higher number of differentially expressed genes enriched in that function. The color gradient from blue to red represents the unadjusted *p*-value.

Based on the analysis of homologous genes, all species selected for analysis contain a single copy of the homologous gene (avoiding paralogous lines). A phylogenetic tree of different smut strains was constructed using a homologous protein-based method (Figure 9A). The results showed that the *Ustilago* genus diverged into two clades approximately 109.2 million years ago (MYA) and *Sporisorium* genus were further diverged from *Ustilago* in about 103 million years ago. Clade analysis revealed that the type strain *Ustilago maydis* is more closely related to *Sporisorium reilianum* than to *Ustilago coicis*, clustering within the same clade. *Ustilago coicis* diverged 21.2 million years ago and clustered with *Ustilago trichophora*, indicating a relatively close evolutionary relationship. The two newly isolated strains are evolutionarily closer to each other. This further confirms that the two isolated strains belong to the genus *Ustilago*, and the results of the gene family evolutionary clustering are consistent with those of the multi-gene phylogenetic tree. Furthermore, during evolution, species undergo gene family expansion and contraction due to selective pressures. By identifying gene families and comparing them with ancestral data on a common timescale, we can determine whether gene families in different species have expanded, contracted, or been lost. We performed gene contraction and expansion analyses on eight selected smut strains and two newly isolated strains. The results are shown in the Figure 9B. Approximately 21 million years ago, most *Ustilago* gueta genes contracted. After this divergence, following the branching of *Ustilago coicis* and *Ustilago trichophora*, these two fungi exhibited significant gene family expansion. The two newly isolated fungi also showed substantial expansion. Enrichment analysis of the expanded genes revealed that they were mainly enriched in membrane-bounded organelles and organonitrogen compound metabolic processes, followed by non-membrane-bounded organelles, intracellular non-membrane-bounded organelles, and regulation of biological processes. KEGG enrichment analysis showed that the largest number of genes were enriched in the spliceosome pathway (Figure 10).

Samples ZC202104 and Z202205 exhibited only gene expansions, with no gene contractions. Since the two samples are very similar, enrichment analysis was conducted solely for ZC202104. The expanded genes in ZC202104 were annotated against the VFDB database (Supplementary Table 4). The results showed that ZCQ1003413, which shares 95.8% sequence identity with the GPA1 gene in *Ustilago maydis*, functions as a guanine nucleotide-binding protein (G protein) involved as a modulator or transducer in various transmembrane signaling systems. Additionally, genes homologous to virulence factors from *Magnaporthe oryzae* and *Ustilago maydis* were identified, suggesting potential cross-host pathogenicity. Functional classification of the expanded virulence genes revealed three dominant categories: catalytic activity (the most abundant, including cytochrome P450s and kinases involved in toxin synthesis and host immune suppression); structural functions (including tubulins and protein complex subunits that mediate the assembly of invasive structures such as hyphae and appressoria); and metabolism/transport (involving genes associated with selenium metabolism and nutrient acquisition critical for host colonization and persistence). Additionally, 14 genes exhibited sequence similarity greater than 90%, with homologs primarily from *Ustilago maydis* and *Candida albicans*. Among these, the CHS5 gene plays a major role in cell wall biogenesis. It is required for the proper morphology of yeast-like cells and is involved in mating tube and dikaryotic hyphae formation. This gene is essential for pathogenicity and may also explain the yeast-like morphological characteristics of ZC202104.

## 4 Discussion

The subphylum *Ustilaginomycotina* comprises about 1,500 species of basidiomycetous plant parasites, with over 600 known species that develop sori in the inflorescences, leaves, or stems of their hosts (Piepenbring, 2003). Common *Ustilaginaceae* fungi include the following predominant strains: *Sphacelotheca reiliana*, *Sphacelotheca sorghi*, *Sporisorium reilianum*, *Ustilago crameri*, *Ustilago esculenta*, *Ustilago hordei*, *Ustilago maydis*, *Ustilago coicis*, *Tilletia horrida* and *Tilletia indica* (Wang et al., 2022). Currently, there was a wealth of reports and extensive research on *Ustilago maydis* and *Sporisorium reilianum*. *Ustilago maydis* which serve as widely used model organisms for studying plant pathogenesis and also being the first basidiomycete to have its complete genome sequenced (Kahmann and Kämper, 2004). Li et al. (2016) conducted an in-depth analysis of the characteristics and key genes involved in the parasitic process of *Ustilago maydis*, proposing 7 distinct stages during host infection, and investigated the crucial factors responsible for reprogramming host tissues into enlarged plant tumors by elucidating differential regulation mechanisms involving hormones, glucose metabolizing enzymes, and transporters between *Ustilago maydis* and *Zea mays* L. Conversely, studies on adlay smut were limited. It is known that *Ustilago coicis* can infect both ovaries and leaves of adlay, leading to adlay smut development (Zhang et al., 2012). Current research primarily focused on field prevention and control measures, neglecting systematic isolation and identification methods for adlay smut strains. Additionally, there was a lack of research on genetic differentiation within this smut strains, as well as developmental patterns and dissemination mechanisms.

In this paper, the color and morphology of coix teliospores were observed, filled the morphological gap in our understanding of these teliospores under a scanning electron microscope. Additionally, two strains that belong to the family of *Ustilaginaceae* were isolated and identified from adlay smut involucres using morphology combined with multi-gene sequencing. These two strains exhibited noticeable morphological differences. Strain ZC 202104 displayed white colonies with prominent yeast-like folds, whereas strain ZC 202205 lacked folds altogether but featured translucent mycelial edges. Better yet, the morphological description of strain ZC 202205 in *Ustilago coicis* had not been previously reported. The results of the isolation and identification indicating the diversity in fungal communities of adlay smut which was consistent with previous reports of metagenomic sequencing and taxonomic analysis of adlay smut (Li et al., 2021). The comprehension of the developmental pattern of coix teliospores could establish a fundamental basis for elucidating the infection mechanism of adlay smut. Our results demonstrated that under optimal temperature and humidity conditions, the teliospores of adlay smut germinated and produced promycelium with a distinct diaphragm. Basidiospores were then produced at the top and sides of the promycelium, and continued to develop into slender mycelium after detaching from the promycelium. Detached basidiospores continued to grow through asexual reproduction by fusing nuclei from individuals with identical mating types or through sexual reproduction by fusing heterogeneous nuclei to form dikaryotic hyphae. Based on this analysis, it is speculated that under favorable nutrient levels and temperature conditions as well as encountering a host plant, the basidiospores could generate dikaryotic hyphae for sexual reproduction, and this process will lead to the infection of the host and the formation of dormant teliospores. The germination process observed by us exhibited similarities to the teliospore of maize as described by Xia et al. (2021), and the germination mechanism of *Tilletia cotroversa* in wheat studied by Wilcoxson and Saari (1996). However, it should be noted that different strains may exhibit variations in growth rates.

Experiments on the biological characteristics of specific strains can provide guidance for subsequent large-scale production and fermentation, and also shed light on the fungus to investigate their adaptability to host plants and environments, we analyzed the carbon and nitrogen utilization and culture conditions of the two isolated *Ustilago* strains. The results showed that the two strains exhibited different morphologies when grown on different nitrogen sources. The optimal carbon source for ZC202104 was glucose and the optimal nitrogen source was peptone, while the optimal carbon source for ZC 202205 was mannose and the optimal nitrogen source was beef paste. Both strains could slow growth in an environment below 18 °C, with an optimum growth temperature of 30 °C, indicating that *Ustilago coicis* infection of adlay was more severe at higher temperatures. Moreover, the pathogen grew best at pH 6.0 in total darkness. Although the fungus is a pathogen, it has the ability to produce various nutritional substances and active compounds. For instance, the infection of *Ustilago esculenta* on *Zizania latifolia* triggered a metamorphosis process, resulting in the development of succulent and aquatic stems with high nutritional content. These transformed plant parts can be consumed as a vegetable (Jose et al., 2016; Li et al., 2023; Wu et al., 2023). Polysaccharide derived from *Ustilago maydis* exhibited significant hepatoprotective effects against acute alcoholic liver injury in mice (Zhang et al., 2020). In addition, *Ustilago maydis* serves as a delectable food and the United States has included *Ustilago maydis* in its list of edible fungi. Moreover, United States offered farmers guidance on cultivation, production, and marketing strategies for *Ustilago maydis* (Shi et al., 2006). In this study, the nutritive components of *Ustilago coicis* were analyzed and demonstrated its high nutritional values. At present, few literature had reported the nutritional value of *Ustilaginaceae* mycelia obtained by liquid culture. Some literatures showed that the nutrient components were riched in the fruiting bodies of *Ustilaginaceae* (Lu et al., 2020). However, it took a long time to obtain fruiting bodies which need to be infected by the host for several months before they can form fruiting bodies. In contrast, liquid culture can rapidly and abundantly produce mycelia, which is more conducive to subsequent development and application. Therefore, to refine the liquid fermentation cultivation of *Ustilago coicis* was much meaningful. Our results revealed that *Ustilago coicis* is rich in nutrients of crude protein, total starch, polysaccharide, and inorganic elements, particularly abundant in various amino acids and vitamins A and C. Polysaccharides were the primary bioactive compounds found in edible fungi which had the activities of against obesity, inflammatory bowel disease, and cancer. Therefore, *Ustilago coicis* might be one of the most promising resources of polysaccharides (Ma et al., 2021). Moreover, our analysis indicates that the isolated strain ZC202104 possesses a high concentration of glutamate, which serves not only as a fundamental component of proteins but also demonstrates a range of physiological activities. While Strain ZC202205 has a high level of lysine, an amino acid that facilitates human growth and development, enhances immune function, and exhibits antiviral properties (Tian and Shi, 2014). As a result, both strains offer their own distinct advantages in amino acid supplementation. Additionally, the strains we isolated are abundant in vitamins A (VA) and C (VC). VA plays a crucial role in growth facilitation as well as maintenance of normal functions in the skin, conjunctiva, cornea etc. The B vitamins contribute to enhanced gastrointestinal peristalsis in the human body by regulating endocrine function and stabilizing the nervous system (Kennedy, 2016). The antioxidant VC possesses remarkable ability to combat free radicals aiding in cancer prevention, lowering cholesterol levels, enhancing immune function, and preventing scurvy. The mycelium of the two strains of *Ustilago coicis* isolated in this study were rich in polysaccharides, amino acids, and vitamins, indicating their potential values for functional food and medicine development.

This study completed the whole-genome sequencing of *Ustilago coicis*, providing valuable data resources for a comprehensive understanding of its infection mechanisms and the molecular basis of its interaction with its host, adlay. The genomes of two isolates, ZC202104 and ZC202205, were approximately 22 Mb in size, with GC contents of 53.83 and 53.75%, respectively, indicating highly conserved genomic features. Gene functional annotation and pathway enrichment analyses were systematically performed by mapping the sequencing data to functional databases such as NR, GO, KEGG, and eggNOG. Within the Gene Ontology (GO) classification, genes involved in “cellular process” and “cellular anatomical entity” were the most abundant, suggesting that *Ustilago coicis* may rely on dynamic remodeling of cellular structures to adapt to the host environment during infection. In particular, functional regulation of cell membrane and nuclear structures may play a key role in its environmental response. KOG analysis revealed significant enrichment of genes involved in “posttranslational modification, protein turnover, chaperones, suggesting that the fungus maintains protein homeostasis through efficient posttranslational modification, folding regulation, and degradation mechanisms. This enables signal transduction and environmental sensing, enhancing its adaptability to host plants. Annotation results from the Carbohydrate-Active Enzyme (CAZy) database revealed that members of the glycoside hydrolase (GH) family were the most numerous, suggesting that *Ustilago coicis* may secrete multiple GH enzymes to degrade major plant cell wall components, such as cellulose and hemicellulose, thereby promoting tissue penetration and colonization. Previous studies have demonstrated that fungal GH family enzymes play a crucial role in pathogenicity. For example, deletion of GH6 and GH7 family cellulase genes in the rice blast fungus (*Magnaporthe oryzae*) reduces its ability to penetrate rice leaf sheaths by up to 70% (Peng et al., 2025), further supporting the central role of GH enzymes in fungal infection. Therefore, it is speculated that the abundant GH genes in *Ustilago coicis* are important molecular tools for its successful infection of adlay. To further identify potential pathogenicity factors, this study conducted effector prediction and virulence-related gene screening. Annotation using the DFVF (Disease-associated Fungal Virulence Factors) database revealed that the two strains were most enriched for virulence factor genes associated with “invasive candidal disease” and “Corn smut.” This suggests that *Ustilago coicis* may share a similar pathogenic strategy with important plant pathogens such as *Ustilago maydis*, potentially interfering with the defense responses of adlay and establishing a parasitic relationship through comparable mechanisms. Gene family clustering and phylogenetic analysis within the *Ustilaginaceae* family indicated that *Ustilago coicis* diverged relatively recently and is most closely related to *Ustilago trichophora*. Notably, analysis of gene family evolutionary dynamics revealed that *Ustilago coicis* exhibited a clear trend of gene expansion with minimal significant contraction. Expanded genes were primarily enriched in the following functional pathways: membrane-bound organelles, organonitrogen compound metabolic processes, and spliceosome- and ribosome-related pathways. Gene expansion analysis of *Ustilago coicis* suggests that it may enhance its ability to infect its host plant by regulating membrane system dynamics. The fungus was significantly enriched in pathways related to organic nitrogen compound metabolism, indicating its capacity to efficiently utilize nitrogen sources, which aids adaptation to the host’s internal nutritional environment. Furthermore, the significant enrichment of ribosome-related genes reflects a state of high protein synthesis, potentially supporting rapid proliferation, formation of infection structures, and expression of numerous effector proteins. In summary, *Ustilago coicis* may have achieved its high adaptability to its specific host plant through a multifaceted genomic adaptation strategy, including the expansion of its cell wall-degrading enzyme repertoire, strengthening of its protein homeostasis regulatory network, and diversification of its effector system. Its extensive evolutionary pattern of gene family expansion, rather than contraction, also suggests that the fungus may possess strong environmental adaptability or the potential to expand its host range. This study provides a solid theoretical and data foundation for subsequent functional gene verification, effector identification, and target screening for disease resistance breeding.

## Data Availability

The datasets presented in this study can be found in online repositories. The names of the repository/repositories and accession number(s) can be found in the article/Supplementary material.

## Footnotes

1
http://www.genome.umd.edu/masurca.html

2
https://github.com/marbl/canu

3
http://mummer.sourceforge.net/

4
https://github.com/isovic/racon

5
https://github.com/broadinstitute/pilon/wiki

6
http://bioinf.uni-greifswald.de/augustus/

7
https://www.ebi.ac.uk/seqdb/confluence/display/THD/GeneWise

8
http://evidencemodeler.github.io/

9
http://www.cbs.dtu.dk/services/RNAmmer/

10
http://lowelab.ucsc.edu/tRNAscan-SE/index.html

11
http://eddylab.org/infernal/

12
http://www.repeatmasker.org

13
https://tandem.bu.edu/trf/home

14
http://orthomcl.org/orthomcl/

15
http://www.ebi.ac.uk/Tools/msa/muscle/

16
http://darwin.uvigo.es/

17
http://www.atgc-montpellier.fr/phyml/

18
http://www.timetree.org/

19
https://github.com/abacus-gene/paml

20
http://sysbio.unl.edu/DFVF/

21
https://effectorp.csiro.au/
