# Correction: Biological characteristics and genetics differentiation of smut fungi in *Coix* L.

**DOI:** 10.3389/fmicb.2026.1858774

**Published:** 2026-04-29

**Authors:** Frontiers Production Office

**Affiliations:** Frontiers Media SA, Lausanne, Switzerland

**Keywords:** adlay smut, biological characteristics, genome sequencing, morphology, nutritional value, teliospores development

An incorrect **Funding** statement was provided.

The correct **Funding** statement reads:

“The author(s) declared that financial support was received for this work and/or its publication. This work was supported by Science and Technology Program in Guizhou Province (Qiankehezhongyindi[2024]006); Guizhou Provincial Key Technology R&D Program ((2022)151); Central Government Funds to Support the reform and Development of local Colleges and Universities ((2023)067); and the Key Laboratory of High Quality, High Efficiency, and Yield Enhancement in Grain and Oil Crops (Qian-Ke-He-Platform ZSYS[2025]037).”

The original version of this article has been updated.

