# Supplementary material for: Biological characteristics and genetics differentiation of smut fungi in Coix L
Source: Front Microbiol. 2026 Apr 17;17:1746178. doi: 10.3389/fmicb.2026.1746178 (PMC13169068; doi:10.3389/fmicb.2026.1746178)
Supplement: Supplementary file 1 [file Supplementary_file_1.docx]

Supplementary Material

# Supplementary Figures and Tables

## Supplementary Figures


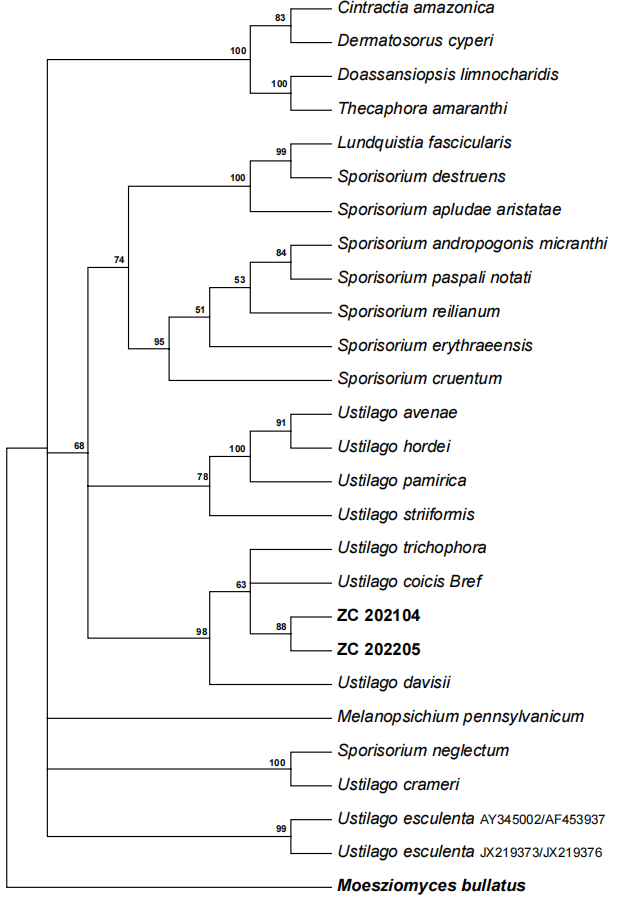


**Supplementary Figure 1.** Phylogram generated from parsimony analysisbased on combined ITS、LSU and ATP6 sequences in *Ustilago* spp. and *Sporisorium* spp. Bootstrap values ≥50% were shown above or below branches


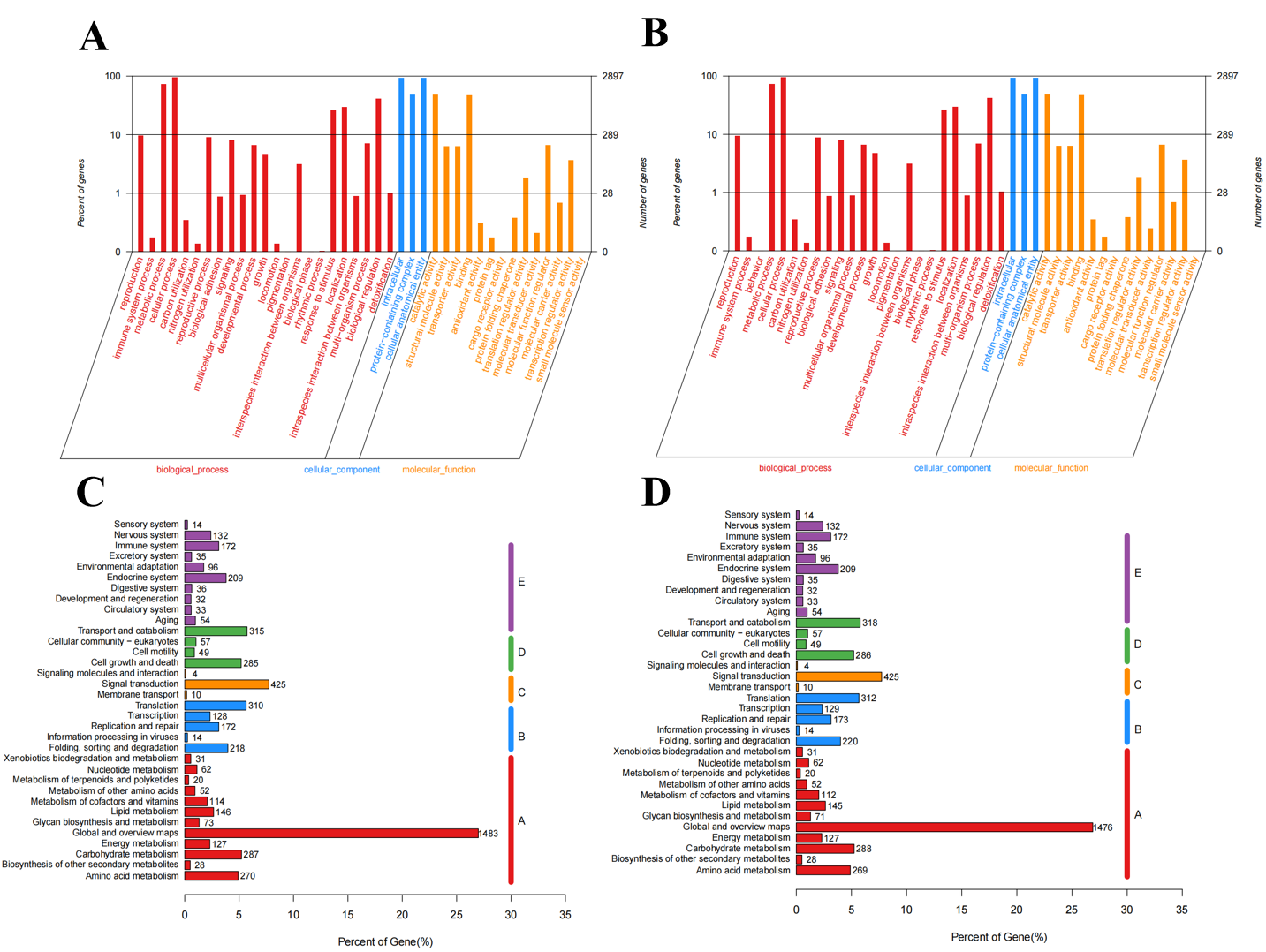


**Supplementary Figure 2**. GO functional annotation and KEGG functional annotation of the genome (A, C) correspond to ZC 202104, and (B, D) correspond to ZC 202205.


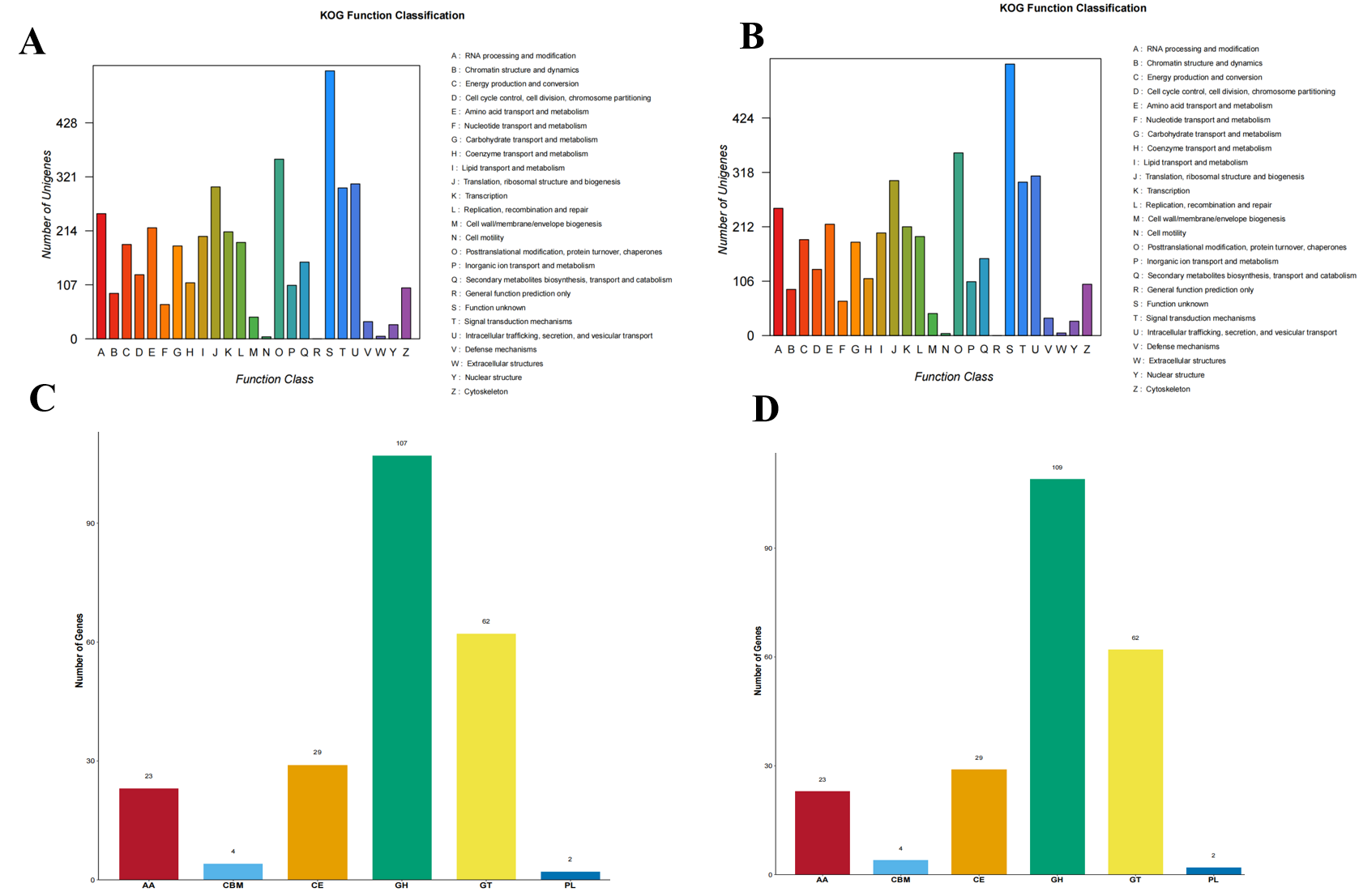


**Supplementary Figure 3.** KOG functional annotation and CAZY functional annotation of the genome. (A, C) correspond to ZC 202104, and (B, D) correspond to ZC 202205. GH: Glycoside hydrolases, CBM: Carbohydrate-binding modules, AA: Auxiliary activities; CE: Carbohydrate esterases, PL: Polysaccharide lyases, GT: Glycosyl transferases.


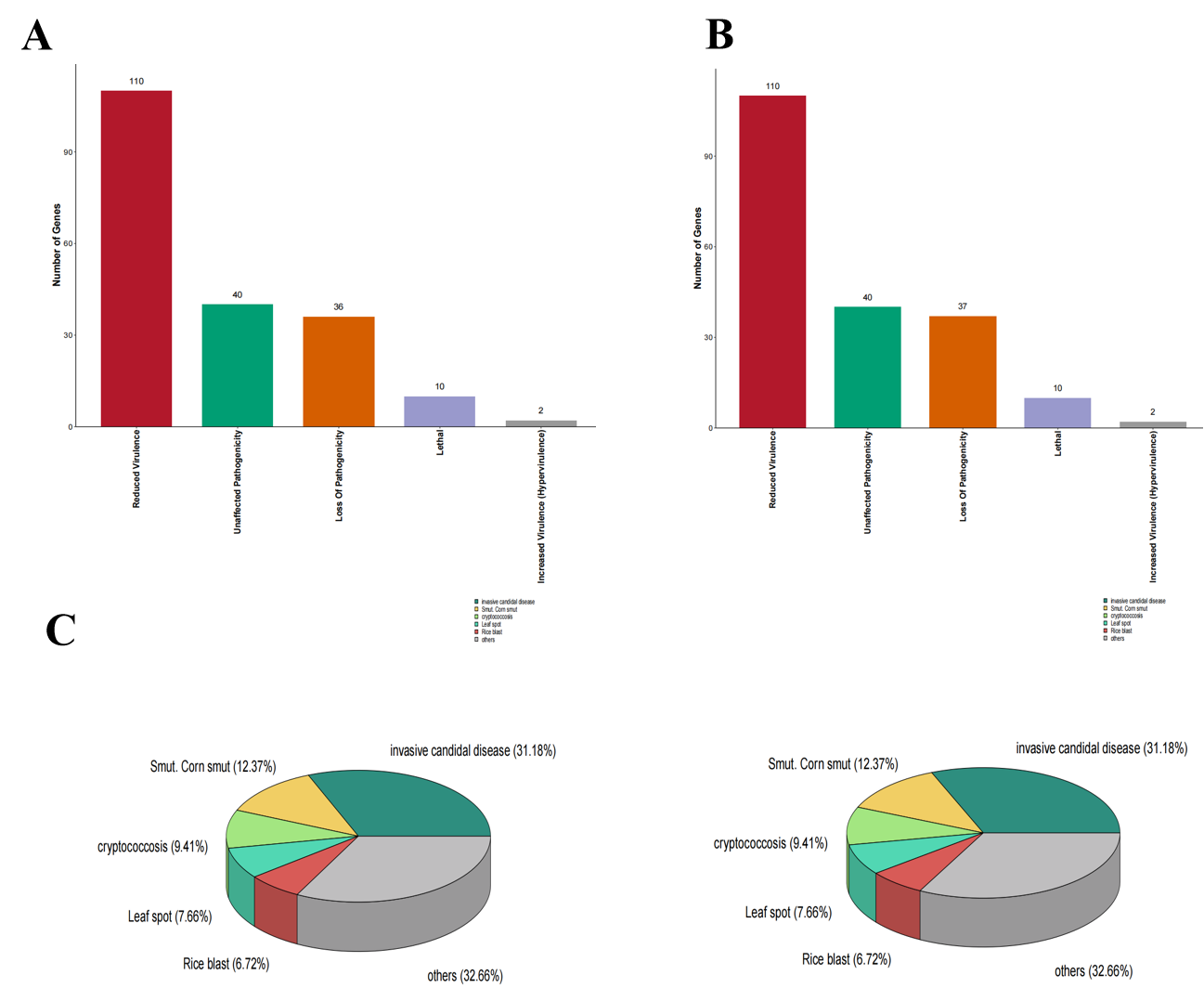


**Supplementary Figure 4**. Analysis of pathogen and host interaction and DFVF functional annotation of the genome. (A, C) correspond to ZC 202104, and (B, D) correspond to ZC 202205.

# 1.2 Supplementary Tables

**Supplementary Table 1** Reference sequences for *Ustilago spp*. and Sporisorium spp. retrieved from GenBank that were included in the phylogenetic trees analyses

| **Species** | **Host** | **Origin** | **GenBank acc. No.** | | | **Source** |
| --- | --- | --- | --- | --- | --- | --- |
|  |  |  | **ITS** | **LSU** | **ATP6** |  |
| *Cintractia amazonica* | *Rhynchospora barbata(Vahl) Kunth* | Germany | DQ875342 | AJ236142 | DQ883448 | MP 2008  (USJ) |
| *Doassansiopsis*  *limnocharidis* | *Limnocharis flava (L.)*  *Buchenau* | Germany | DQ875344 | AF009850 | DQ883452 | HUV 15198 |
| *Dermatosorus cyperi* | *Cyperus cellulloso-*  *reticulatus Bo¨ckeler* | Germany | DQ875343 | AJ236157 | DQ883450 | HUV 15991 |
| *Lundquistia*  *fascicularis* | *Digitaria brownie* | Australia | AY740035 | AY740088 | - | 58832a  (DAR) |
| *Melanopsichium*  *pennsylvanicum* | *Polygonum glabrum Willd.* | Germany | AY740040 | AY740093 | DQ883458 | HUV 17548 |
| *Moesziomyces bullatus* | *Paspalum distichum* | India | AY740153 | - | - | Ust. Exs.  833 (M) |
| *Thecaphora amaranthi* | *Amaranthus hybridus L.* | Germany | - | AF009873 | DQ883465 | HUV 15882 |
| *Sporisorium andropogonis-micranthi* | *Capillipedium*  *spicigerum* | Australia | AY740047 | AY740100 | - | 56595 (M) |
| *Sporisorium apludae-*  *aristatae* | *Apluda mutica* | India | AY740045 | AY740098 | - | 56590 (M) |
| *Sporisorium cruentum* | *Sorghum halepense* | USA | AY344974 | AF453939 | - | Ust. Exs.  687 (M) |
| *Sporisorium destruens* | *Panicum miliaceum* | Romania | AY344976 | AY747077 | - | Ust. Exs.  472 (M) |
| *Sporisorium*  *erythraeensis* | *Hackelochloa*  *granularis (L.) Kuntze* | India | AY740049 | AY740102 | DQ883462 | Ust. Exs.  849 (M) |
| *Sporisorium neglectum* | *Setaria pumila* | Germany | AY740056 | AY740109 | - | RB 2056  (TUB) |
| *Sporisorium paspali-*  *notati* | *Paspalum notatum* | Cuba | AY344982 | AF453944 | - | Stoll *et al*.,  2005 |
| *Sporisorium reilianum* | *Zea mays L.* | Germany | - | AF453942 | DQ883463 | Lu¨bberstedt, s.n. |
| *Ustilago avenae* | *Arrhenaterum elatius (L.) J. & K. Presl* | Germany | AY740062 | AY740116 | DQ883466 | RB 3092  (TUB) |
| *Ustilago coicis* | *Coix lacryma-jobi* | China | JX219371 | JX219374 | - | Zhang *et al*.,  2013 |
| *Ustilago crameri* | *Setaria italica* | India | AY344999 | AY740143 | - | Ust. Exs.  995 (M) |
| *Ustilago davisii* | *Glyceria multiflora Stena* | Germany | AY740169 | DQ875374 | DQ883468 | HUV 19252 |
| *Ustilago esculenta* | *Zizania latifolia* | China | JX219373 | JX219376 | - | Zhang *et al*.,  2013 |
| *Ustilago esculenta* | *Zizania latifolia* | Taiwan,  China | AY345002 | AF453937 | - | Ust. Exs.  590 (M) |
| *Ustilago hordei* | *Avena sativa* | Spain | AY740068 | AY740122 | - | F 947/GD  1300 |
| *Ustilago pamirica* | *Bromus gracillimus* | Iran | AY345005 | AY740145 | - | Ust. Exs.  789 (M) |
| *Ustilago striiformis* | *Alopecurus pratensis L* | Germany | AY740172 | DQ875375 | DQ883469 | HUV 18286 |
| *Ustilago trichophora* | *E. colona* | India | AY740073 | AY740125 | - | 56564 (M) |
| ZC202104 | *Coix lacryma-jobi* | China | **OQ283646** | **OQ283704** | **OQ417285** | In this study |
| ZC202205 | *Coix lacryma-jobi* | China | **OQ283647** | **OQ283705** | **OQ417286** | In this study |

**Note:** The accession numbers marked in bold face refer to sequences new in this study. Voucher abbreviations: -: Without accession numbers; M: München, Germany; TUB: Tübingen, Germany; Ust. Exs.: Ustilaginales Exsiccata; GD: Günter Deml; F: Franz Oberwinkler; H.U.V.: Herbarium Ustilaginales Vánky; RB: Robert Bauer; MP: Meike Piepenbring.

**Supplementary** **Table 2.** Amino acid content of two strains of *Ustilago coicis*(±s, n=3)

| **Amino acids** | **Content(（ug/g)** | | **Amino acids** | **Content(（ug/g)** | |
| --- | --- | --- | --- | --- | --- |
|  | **ZC 202104** | **ZC 202205** |  | **ZC 202104** | **ZC 202205** |
| Arg | 44.49±0.42 | 40.93±0.56 | Pro | 18.99±0.12 | 19.40±0.12 |
| Lys＊ | 44.47±0.42 | 61.43±0.31 | Ala | 35.79±0.32 | 41.96±0.36 |
| Phe＊ | 20.76±0.54 | 25.14±0.47 | Thr＊ | 22.22±0.60 | 24.99±0.48 |
| Leu＊ | 46.04±0.53 | 54.16±1.44 | Ser | 33.60±0.23 | 35.91±0.31 |
| Trp＊ | 0.21±0.00 | 0.22±0.00 | Gly | 29.17±0.47 | 31.19±0.50 |
| ILe＊ | 28.29±0.45 | 30.21±0.47 | Gln | 0.09±0.00 | 0.66±0.00 |
| Met＊ | 16.37±0.33 | 32.65±0.31 | Asp | 20.51±0.33 | 19.26±0.15 |
| Tyr | 24.45±0.73 | 29.83±0.53 | Glu | 56.99±0.45 | 44.55±0.25 |
| Cys | 1.12±0.01 | 0.42±0.01 | His | 17.75±0.29 | 17.89±0.11 |
| Val＊ | 31.86±0.29 | 43.44±0.42 | Asn | 0.57±0.00 | 0.50±0.01 |
| Total amino-  acids | 493.74 | 554.74 | Essential amino-acids | 210.22 | 272.24 |
| Total of non-  essential amino-acids | 283.52 | 282.5 | Essential amino-acids /Total amino-acids | 42.58% | 49.08% |

**Note:＊ is** **essential amino-acid.**

**Supplementary Table 3.** Combined effectors protein comparison table

| **Protein Category** | **ZC202104** | **ZC 202205** | **ZC202104**  **Proportion** | **ZC 202205**  **Proportion** | **Notes** |
| --- | --- | --- | --- | --- | --- |
| Total Protein Count | 5,873 | 5,869 | - | - |  |
| Cytoplasmic effector | 1,407 | 1,413 | 23.96% | 24.08% |  |
| └ High-confidence Cytoplasmic Effectors | 437 | 418 | - | - | confidence score ≥ 0.8 |
| Apoplastic effector | 77 | 75 | 1.31% | 1.28% |  |
| Non-effector | 4,339 | 4,333 | 73.88% | 73.83% |  |
| Total Effectors | 1,484 | 1,488 | 25.27% | 25.35% | Cytoplasmic + Periplasmic Effectors |

**Supplementary Table 4.** Annotation results of expanded genes in ZC202104 against VFDB database

| **GeneID** | **Identity%** | **IdentityLen** | **Gene**  **Symbol** | **Organism** | **Description** | **Disease** |
| --- | --- | --- | --- | --- | --- | --- |
| ZCQ1003068.1 | 99 | 192 | RAS2 | *Ustilago maydis* | Unknown | Smut. Corn smut |
| ZCQ1004759.1 | 98.8 | 340 | NULL | *Ustilago maydis* | Catalytic activity: ATP + a protein = ADP + a phosphoprotein. | Smut. Corn smut |
| ZCQ1003987.1 | 98.7 | 228 | UBI4 | *Candida albicans* | Unknown | invasive candidal disease |
| ZCQ1004880.1 | 98.7 | 76 | UBI4 | *Candida albicans* | Unknown | invasive candidal disease |
| ZCQ1004909.1 | 98.7 | 152 | UBI4 | *Candida albicans* | Unknown | invasive candidal disease |
| ZCQ1003713.1 | 96.6 | 354 | KPP2 | *Ustilago maydis* | Catalytic activity: ATP + a protein = ADP + a phosphoprotein. | Smut. Corn smut |
| ZCQ1003413.1 | 95.8 | 353 | GPA1 | *Ustilago maydis* | Function: Guanine nucleotide-binding proteins (G proteins) are involved as modulators or transducers in various transmembrane signaling systems. | Smut. Corn smut |
| ZCQ1002203.1 | 95.8 | 542 | UKC1 | *Ustilago maydis* | Catalytic activity: ATP + a protein = ADP + a phosphoprotein. | Smut. Corn smut |
| ZCQ1002413.1 | 92.2 | 591 | CRU1 | *Ustilago maydis* | Unknown | Smut. Corn smut |
| ZCQ1000776.1 | 91.8 | 1494 | CHS5 | *Ustilago maydis* | Function: Plays a major role in cell wall biogenesis. Required for the proper morphology of yeast-like cells. Involved in mating tube and dikaryotic hyphae formation. Essential for pathogenicity. | Smut. Corn smut |
| ZCQ1000099.1 | 91.7 | 241 | ERG2 | *Ustilago maydis* | Function: Catalyzes the reaction which results in unsaturation at C-7 in the B ring of sterols. | Smut. Corn smut |
| ZCQ1002414.1 | 91.6 | 131 | HIS3 | *Fusarium longipes* | Subunit: The nucleosome is a histone octamer containing two molecules each of H2A, H2B, H3 and H4 assembled in one H3-H4 heterotetramer and two H2A-H2B heterodimers. The octamer wraps approximately 147 bp of DNA (By similarity). | seedling collar rot |
| ZCQ1000150.1 | 91.4 | 590 | UM01886.1 | *Ustilago maydis* | Caution: The sequence shown here is derived from an EMBL/GenBank/DDBJ whole genome shotgun (WGS) entry which is preliminary data. | Smut. Corn smut |
| ZCQ1000730.1 | 90.2 | 357 | GPA2 | *Ustilago maydis* | Function: Guanine nucleotide-binding proteins (G proteins) are involved as modulators or transducers in various transmembrane signaling systems. | Smut. Corn smut |
